# EPR of Photoexcited Triplet-State Acceptor Porphyrins

**DOI:** 10.1021/acs.jpcc.1c03278

**Published:** 2021-05-19

**Authors:** Ashley J. Redman, Gabriel Moise, Sabine Richert, Erin J. Viere, William K. Myers, Michael J. Therien, Christiane R. Timmel

**Affiliations:** † Centre for Advanced Electron Spin Resonance (CÆSR), University of Oxford, South Parks Road, Oxford OX1 3QR, United Kingdom; ‡ Institute of Physical Chemistry, University of Freiburg, Albertstraße 21, 79104 Freiburg, Germany; § Department of Chemistry, Duke University, French Family Science Center, 124 Science Drive, Durham, North Carolina 27708, United States

## Abstract

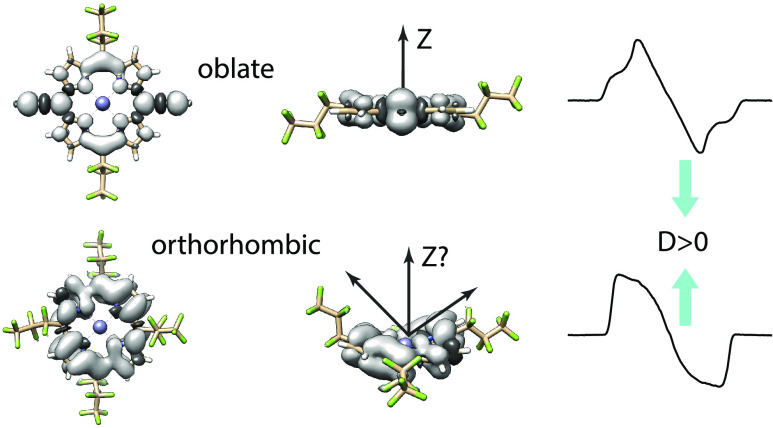

The photoexcited triplet states of porphyrin architectures are of significant interest in a wide range of fields including molecular wires, nonlinear optics, and molecular spintronics. Electron paramagnetic resonance (EPR) is a key spectroscopic tool in the characterization of these transient paramagnetic states singularly well suited to quantify spin delocalization. Previous work proposed a means of extracting the absolute signs of the zero-field splitting (ZFS) parameters, *D* and *E*, and triplet sublevel populations by transient continuous wave, hyperfine measurements, and magnetophotoselection. Here, we present challenges of this methodology for a series of *meso*-perfluoroalkyl-substituted zinc porphyrin monomers with orthorhombic symmetries, where interpretation of experimental data must proceed with caution and the validity of the assumptions used in the analysis must be scrutinized. The EPR data are discussed alongside quantum chemical calculations, employing both DFT and CASSCF methodologies. Despite some success of the latter in quantifying the magnitude of the ZFS interaction, the results clearly provide motivation to develop improved methods for ZFS calculations of highly delocalized organic triplet states.

## Introduction

Porphyrin molecules are well established as ideal building blocks for molecular wires in nanoscale electronic devices, spintronics, and photovoltaic cells.^1−10^ Despite the extensive research dedicated to the understanding of their ground and excited-state properties and their potential applications, porphyrin systems remain an elusive class of organic compounds, especially when it comes to their magnetic properties. As a result, they also represent an ideal testing ground for numerous computational and quantum chemical studies in conjunction with spectroscopic techniques such as transient and pulse electron paramagnetic resonance (EPR), electronic absorption, and fluorescence.^11−19^

The targeted design of porphyrin molecular devices requires the ability to control the spatial distribution of the spin density within a multiporphyrin array. *Meso*-perfluoroalkyl substitution provides a significant modulation with respect to the classic 5,10,15,20-tertraphenylporphyrin electronic structure.^20−22^ These non-π-conjugating, strongly σ-electron-withdrawing substituents stabilize the frontier orbitals in (porphinato)metal structures. Potentiometric and TD-DFT electronic structure studies established that the HOMOs and LUMOs of *meso*-(perfluoroalkyl)porphyrins are uniformly lowered by ∼0.15 eV by each such substitution relative to an analogous meso-aryl porphyrin. As a case in point, the *E*_1/2_^0/+^ and *E*_1/2_^–/0^ values measured for [5,10,15,20-tetrakis-(heptafluoropropyl)-porphinato]zinc(II) (**A**_**4**_) are each 0.67 eV stabilized relative to those determined for the [5,10,15,20-tetraphenylporphinato]zinc(II) benchmark (TPPZn).^21^

**Figure 1:**
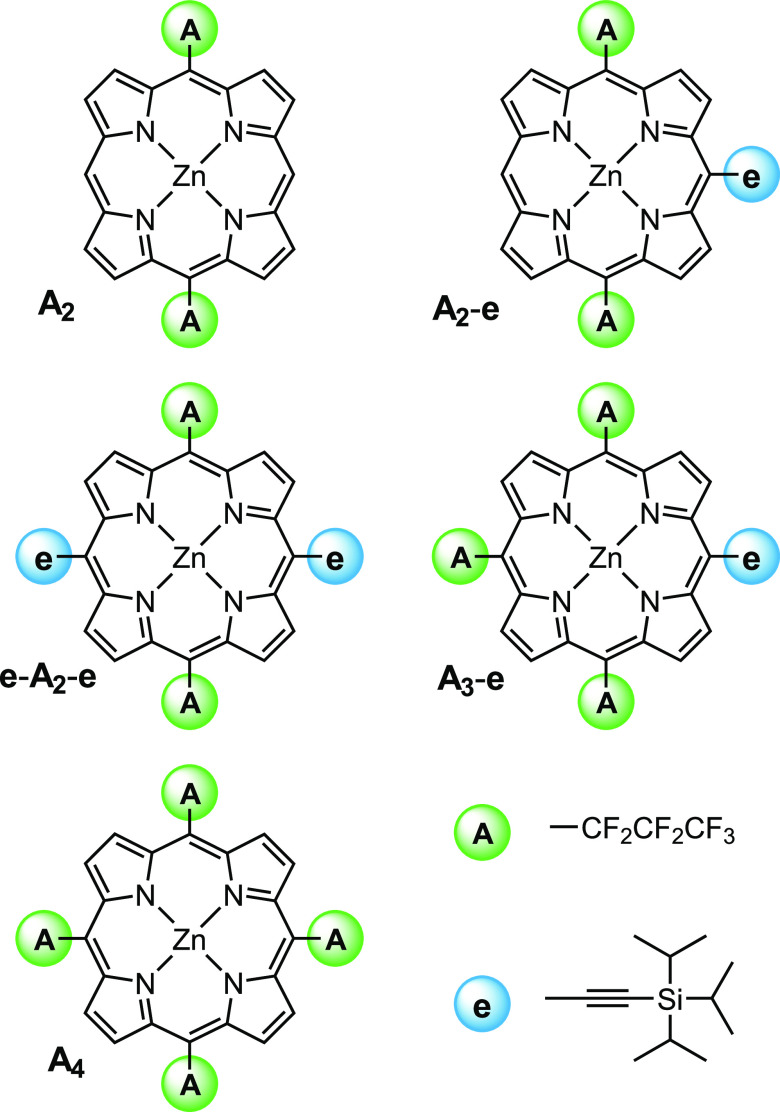
Chemical structures of the acceptor-substituted porphyrins (denoted **A**_**2**_, **A**_**2**_-**e**, **e-A**_**2**_-**e**, **A**_**3**_-**e**, and **A**_**4**_) investigated in this work.

Previous EPR studies have been successful in revealing the extensive electronic communication between the subunits of linear and cyclic oligoporphyrin wires in their radical cation^23−26^ or anion^27^ states, as well as their photoexcited triplet states.^28−32^ In this contribution, we focus on the magnetic fine-structure parameters of a series of perfluoroalkyl meso-substituted porphyrin monomers. In particular, we would like to establish whether small systematic changes in the number and type of acceptor groups around the porphyrin core are sufficient to realize observable changes in the triplet spin-density distributions. The systems in Figure 1 are investigated using multifrequency time-resolved/transient EPR (trEPR) and pulse electron–nuclear double resonance (ENDOR) spectroscopy. The experimental findings are reconciled with the results of quantum chemical calculations employing DFT and complete active space self-consistent field (CASSCF) methodologies.

## Methods

### Sample Preparation

Solutions of the acceptor-substituted porphyrin monomers at a concentration of approximately 0.2 mmol dm^–3^ in 2-methyltetrahydrofuran (2-MeTHF) (Sigma-Aldrich) were prepared. Prior to preparation, the inhibitor-free solvent was passed through a column of activated aluminum oxide. The solutions were transferred either to a (i) 3.8 mm OD, 2.7 mm ID, (ii) 1.6 mm OD, 1.1 mm ID, or (iii) 0.9 mm OD, 0.5 mm ID quartz tube and flash-frozen in liquid nitrogen prior to the EPR measurements at X-, Q-, or W-band frequencies, respectively.

### EPR Measurements

#### Laser Excitation

Laser excitation at 532 nm, unless otherwise stated, was performed using an Opotek Opolette HE355 OPO pumped by the third harmonic of a Nd:YAG laser. The excitation energies were 2–3 mJ for X-/Q-band measurements and 0.6 mJ for W-band measurements, each at a repetition rate of 20 Hz. The laser power was controlled through a combination of a λ/2 waveplate and a Glan–Taylor polarizer. After the last laser turning mirror, the light was depolarized using an achromatic depolarizer. For X-/Q-band measurements, the light was directed through the cryostat optical window, whereas for the W-band, the laser beam was directed to the top of the sample via an optical fiber within the EPR tube.

#### Transient EPR Measurements

All transient EPR experiments were performed on a Bruker ELEXSYS-I E680 spectrometer at 20 K. The temperature was controlled using a helium gas-flow cryostat and an ITC temperature controller from Oxford Instruments. The X-band measurements were performed using a critically coupled EN 4118X-MD4-W1 resonator, whereas the W-band measurements used an EN 600-1021H TeraFlex resonator. The trEPR measurements were performed in direct detection mode using the transient recorder with a microwave power of 0.2−2 mW. Typically, the static magnetic field sweep width was 120 mT (256 points). For each field position, a time trace with 4096 points and 100 transient averages was recorded using a time base of 2 ns. A video amplifier bandwidth of 20 MHz was used for the X-band, while time resolution is limited to ca. 200 ns by the resonator. While the X-band measurements used DC-AFC, the W-band data were collected using AC-AFC and a Stanford Research 560 voltage preamplifier with a 3–200 kHz bandpass.

The background signal was removed by a linear 2D baseline correction using: (i) the signal acquired before the laser pulse and (ii) the high- and low-field off-resonant transients. The corrected spectra were integrated over a 0.8 μs time gate centered at 0.6 μs after the laser pulse. Within this range, no significant change in the signal shape or intensity was observed. W-band data required up to 20° phase shift via a Hilbert transform.

#### Magnetophotoselection Measurements

The magnetophotoselection measurements were performed in line with the X-band trEPR protocol, with a few significant modifications. A second polarizer was placed after the last turning mirror, in place of the depolarizer, to generate linearly polarized light, aligned vertically or horizontally relative to the applied magnetic field. The excitation wavelength was altered for different experiments and the value chosen is reported alongside the data. Care was taken to achieve a constant laser power between polarizations at a given wavelength.

#### Pulse EPR Measurements

The pulse EPR measurements were performed at 20 and 5 K using a Bruker ELEXSYS-I E680 spectrometer for X-/W-band frequencies using the same resonator as for the trEPR experiments and a Bruker ELEXSYS-II E580 spectrometer for Q-band frequencies using an EN 5107D2 resonator.

Mims ENDOR measurements were performed with the pulse sequence: laser pulse–π/2–τ–π/2–*T*–π/2–τ-echo. The first π/2 microwave (mw) pulse was placed 600 ns after the laser pulse. A radio frequency (rf) pulse applied during the delay period *T* had lengths of 15, 24, and 42 μs at X-, Q-, and W-bands, respectively. The rf pulse power and length were optimized using nutation experiments of the ^1^H Larmor frequency signal to correspond to a π pulse. The mw pulse lengths were *t*_π/2_ = 16, 52, and 52 ns for X-, Q-, and W-bands, respectively. During the experiment, the rf frequency was stochastically varied over a sweep width of 16 MHz centered at the ^1^H Larmor frequency with 321 linearly equally spaced points. The spectra displayed are the results of ca. 100 accumulations with one shot per point and a shot repetition time dictated by the laser. At each magnetic field position, ENDOR spectra were acquired for several τ values (typically 120, 180, and 240 ns). The spectra reported herein arise from a summation of the individual τ spectra.

### Simulations and Computational Details

#### Spectral Simulations

The trEPR spectra were simulated in MATLAB version 9.5 (R2018b) as absorption powder spectra using full matrix diagonalization via the pepper function from the EasySpin package.^33^ The magnetophotoselection spectra were simulated using an in-house routine; further details are reported in the SI.

#### Quantum Chemical Calculations

All property calculations were performed using the ORCA 4.1.2 program.^34,35^ Single-point energy and EPR property calculations for the excited triplet state were performed on structures optimized by DFT. Ground singlet-state optimizations were performed in Turbomole 6.1 under *C*_1_ symmetry, using the B3LYP functional and the def2-TZVP basis set in combination with the RI approximation.^36^ A second optimization to the triplet state with B3LYP/def2-TZVP without symmetry constraints was performed in ORCA.

EPR parameter calculations were performed with B3LYP and the 6-31G(d) basis set for zinc and EPR-II for all remaining nuclides. For the zero-field splitting parameters, only the spin–spin contribution was considered using the spin-unrestricted natural orbital (UNO) approach.

EPR parameters were also calculated using CASSCF, considering an active space of four electrons and four orbitals, CAS(4,4), using the def2-SVP basis set and def2-TZVPP for zinc. The initial active space orbitals were selected from an initial restricted open-shell Hartree–Fock (ROHF) calculation.

## Results and Discussion

### Transient Continuous Wave EPR

**Figure 2:**
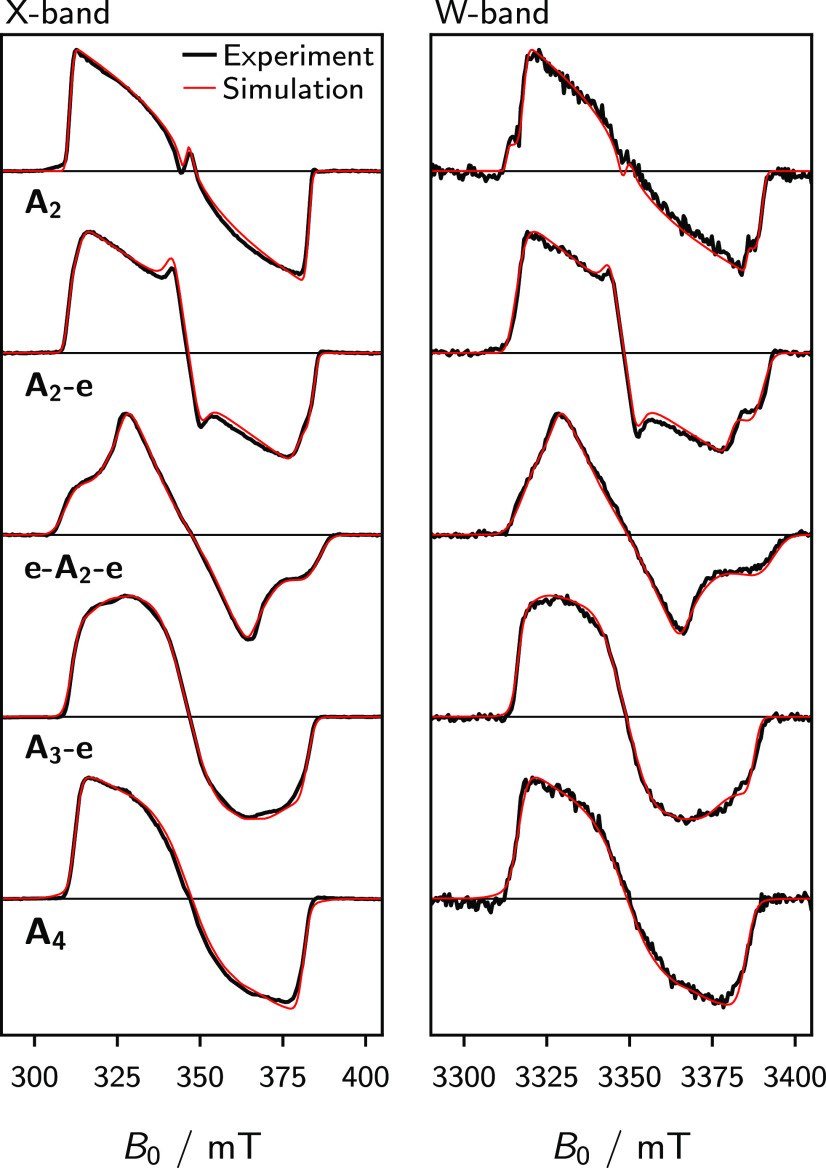
Experimental frozen-solution trEPR spectra of **A**_**2**_, **A**_**2**_**-e**, **e-A**_**2**_**-e**, **A**_**3**_**-e**, and **A**_**4**_ (black) recorded at 20 K at X- and W-band microwave frequencies after photoexcitation at 532 nm. The spectral simulations obtained using the parameters in Table 1 are shown in red. Note: the experimental spectra are the average obtained over a 0.8 μs time window following the laser pulse.

Figure 2 shows the frozen-solution trEPR spectra of the acceptor porphyrin systems measured at X-band (≈9.75 GHz) and W-band (≈94 GHz) microwave frequencies following photoexcitation with a laser pulse at a wavelength of 532 nm. In many respects, the spectral signature of these acceptor porphyrins seems typical for a photogenerated porphyrin triplet state.^37,38^ The sequence of absorptive (*a*) and emissive (*e*) features in the electron-spin polarization pattern indicates a non-Boltzmann population of the three magnetic sublevels. This spin-polarized triplet state originates from differences in the relative rates of intersystem crossing (ISC) and relaxation to/from the different manifolds of the triplet state. Furthermore, the turning points/peak positions in these spectra are dictated by two magnetic fine-structure interaction parameters: the **g**- and **D**-tensors. The former parameter is a measure of the strength and anisotropy of the Zeeman interaction between the electron spin and the external magnetic field. The latter, termed the zero-field splitting (ZFS) interaction, is only present in systems with more than one unpaired electron. In these systems, this interaction is dominated by the magnetic dipolar (spin–spin), through space interaction between the (two) electrons that comprise the triplet.

The ZFS interaction can, in principle, be used to determine the extent of delocalization of the triplet spin density.^28,29^ Assuming the validity of the point dipole approximation and provided a suitable reference frame is chosen, the spin–spin contribution to the elements of the **D**-tensor is completely specified by two independent parameters
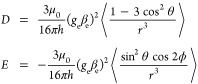
1where *r* is the interspin distance, θ is the polar angle between the interspin vector and the (molecular) *z*-axis, and ϕ is the corresponding azimuthal angle in the *xy*-plane.^39,40^ The angular brackets in eq 1 indicate an expectation value taken over the triplet wave function. Therefore, the magnitudes of the *D* and *E* parameters are intrinsically linked to the average interspin distance and the orthorhombicity of the spin density, respectively. Contemporary investigations employing trEPR and ENDOR techniques have shown that the sign of the ZFS *D* parameter is crucial in the interpretation of the extent of spin delocalization in the photoexcited triplet states of porphyrin molecules.^28,29^ A positive *D*-value indicates that the *Z*-axis of the **D**-tensor is parallel to the molecular *z*-axis (perpendicular to the porphyrin plane), whereas a negative *D*-value indicates that the ZFS *Z*-axis is in the plane of the porphyrin. As a direct consequence of the link between the sign of *D* and the orientation of the **D**-tensor, the symmetry of the spin density can be described as follows: a *D* > 0 implies an “oblate” spin density with respect to the porphyrin structure, while *D* < 0 implies a “prolate” spin density.^41^ However, as will be shown in this work, there is another aspect to this criterion, which has not been addressed with regards to porphyrin systems until now. Namely, for significantly orthorhombic spin densities, observed in the molecules considered here, the interpretation of the orientation and sign of the ZFS tensor/parameters becomes significantly more complex.

The fine-structure parameters described above, **g**, *D*, and *E*, are extracted from the experimental X- and W-band frequency trEPR spectra (black traces in Figure 2) via a global numerical fitting using the EasySpin package in MATLAB.^33^ The electron-spin polarization of the triplet state is accounted for in the numerical simulations by a set of three values, *P_X_*, *P_Y_*, and *P_Z_*, which describe the relative populations for the eigenstates of the triplet in the absence of an external magnetic field. The eigenstates of this zero-field Hamiltonian are labeled *X*, *Y*, and *Z* (see the SI for the complete discussion).

The simulated trEPR spectra are shown in red in Figure 2 and the corresponding simulation parameters are presented in Table 1. It is well known that the parametrization of these spin systems from a standard trEPR experiment, via the phenomenological spin Hamiltonian, is not unique: identical simulations can be produced by inverting the sign of the *D*-value and simultaneously redistributing the sublevel populations. For a sign assignment to be possible, additional information is required, often obtained from magnetophotoselection (MPS) or pulse ENDOR measurements.^29,42,43^ Inspection of Table 1 reveals that we have assigned a negative sign to the *D*-value of **A**_**2**_. This assignment for **A**_**2**_ is as unexpected as it is important for the conclusions of this paper. Throughout the remaining discussion, the parameters in Table 1, with particular emphasis on the outlier (**A**_**2**_), will be justified and interpreted.

**Table 1:** trEPR Simulation Parameters:*g*-Values, ZFS Parameters (*D* and *E*), and Relative Sublevel Populations (*P_X_*, *P_Y_*, and *P_Z_*)^a^

	*D*/MHz	*E*/MHz	*g_x_*	*g_y_*	*g_z_*	*P_X_*	*P_Y_*	*P_Z_*
**A_2_**	–1017	+333	2.0033	2.0033	2.0003	0.81	0.00	0.19
**A_2_-e**	+1033	–272	2.0041	2.0038	2.0006	0.10	0.00	0.90
**e-A_2_-e**	+1074	–105	2.0034	2.0040	1.9989	0.00	0.10	0.90
**A_3_-e**	+1000	–214	2.0039	2.0037	2.0017	0.05	0.00	0.95
**A_4_**	+967	–300	2.0035	2.0032	2.0021	0.22	0.00	0.78

a Parameters were extracted from a global simulation of the spectra shown in Figure 2. The sublevel populations have been normalized such that the smallest is zero and the sum equals unity. The assignment of a negative *D*-value for **A**_**2**_ is discussed in detail in the main text. The corresponding simulations of the trEPR and MPS experimental data for a positive *D*-value together with the associated populations are provided in the SI.

For all systems, except **A**_**2**_, the spectra in Figure 2 display the archetypal polarization pattern, *aaaeee*, specific to many zinc porphyrin triplet states.^38^ As described in previous EPR work,^28,29^ the *D*-value of monomeric porphyrin systems has been determined to be positive, and thus this polarization pattern is assigned to the preferential population of the out-of-plane triplet *Z* sublevel, as highlighted by the values in Table 1.

At W-band microwave frequencies, the enhanced *g*-value resolution further facilitates the discussion of the subtle effects observed in these porphyrin systems. The asymmetry of the W-band spectra in Figure 2, relative to the X-band spectra, is indicative of an anisotropic **g**-tensor, which is quantified by the simulated *g*-values reported in Table 1. In a zinc porphyrin monomer, ISC from the excited singlet state into the observable triplet state has been shown to be dominated by a direct spin–orbit coupling interaction, resulting in a preferential population of the out-of-plane triplet manifold, in marked contrast to the in-plane population seen in free-base porphyrin monomers.^44−47^ Since the shifts in the *g*-values are also a result of perturbations due to spin–orbit coupling, we expect a correlation between the sublevel populations and the *g*-values.^46,48,49^ For a typical zinc porphyrin monomer, the *g_z_*-axis and the ZFS *Z*-axis are collinear and perpendicular to the porphyrin plane. As a result, the triplet *Z* sublevel is preferentially populated, as is indeed indicated by the simulation parameters in Table 1 for all systems except **A**_**2**_. For the latter, the observed preferential population of the *X* sublevel is in line with a reorientation of the **D**-tensor (*D*-value sign inversion), such that the *X*-axis is now oriented perpendicular to the porphyrin plane.

Overall, the *D*-values show a modest trend of decreasing magnitude with an increasing number of acceptor groups, whereas the *E*-values display no obvious trend. In systems with such complex electronic symmetries, manifested in the highly orthorhombic ZFS (Table 1) and spin-density distributions (Figure 4), an interpretation of the spin density cannot solely rely on the link between the *D*-value and the point dipole approximation. For example, predictions based purely on the symmetry of the molecule are not sufficient to explain the increase in |*D*| from **A**_**2**_**-e** to **e-A**_**2**_**-e**. Furthermore, predictions of an oblate to prolate symmetry transition, associated with a change in the sign of the *D*-value, should be accompanied by a significant magnitude change (due to the 1–3 cos^2^ θ term in eq 1). However, as we shall outline in our Magnetophotoselection section below and in more detail in the SI, the experimental data point with some clarity toward a negative *D* for **A**_**2**_. Yet, an associated change in |*D*|, as would be expected from the simplistic interpretation above, is clearly not observed (Table 1). The behavior of the *D*- and *E*-values in these molecules seems to be more subtle. It is thus apparent that the highly successful qualitative framework employed in previous work is no longer sufficient to understand these systems.^28,29,31,32^ These complications are most likely a consequence of a more complex symmetry for the spin density in these systems, *vide infra*.

### Magnetophotoselection

We now turn our attention to a more detailed investigation of the sign of *D*. Whilst the sign of the *D*-value is not straightforward to determine for most frozen solution or powder samples, photogenerated triplet states benefit from an internal reference system: the optical transition dipole moments. This optical reference frame can be exploited in a magnetophotoselection (MPS) experiment, whereby polarized light is used to selectively alter the weights of different molecular orientations with respect to the static external magnetic field vector. The orientation selection effect caused by the polarization of the laser light (parallel/perpendicular to the external field) causes changes to the intensities of the EPR transitions.^29,42,43,50^

**Figure 3:**
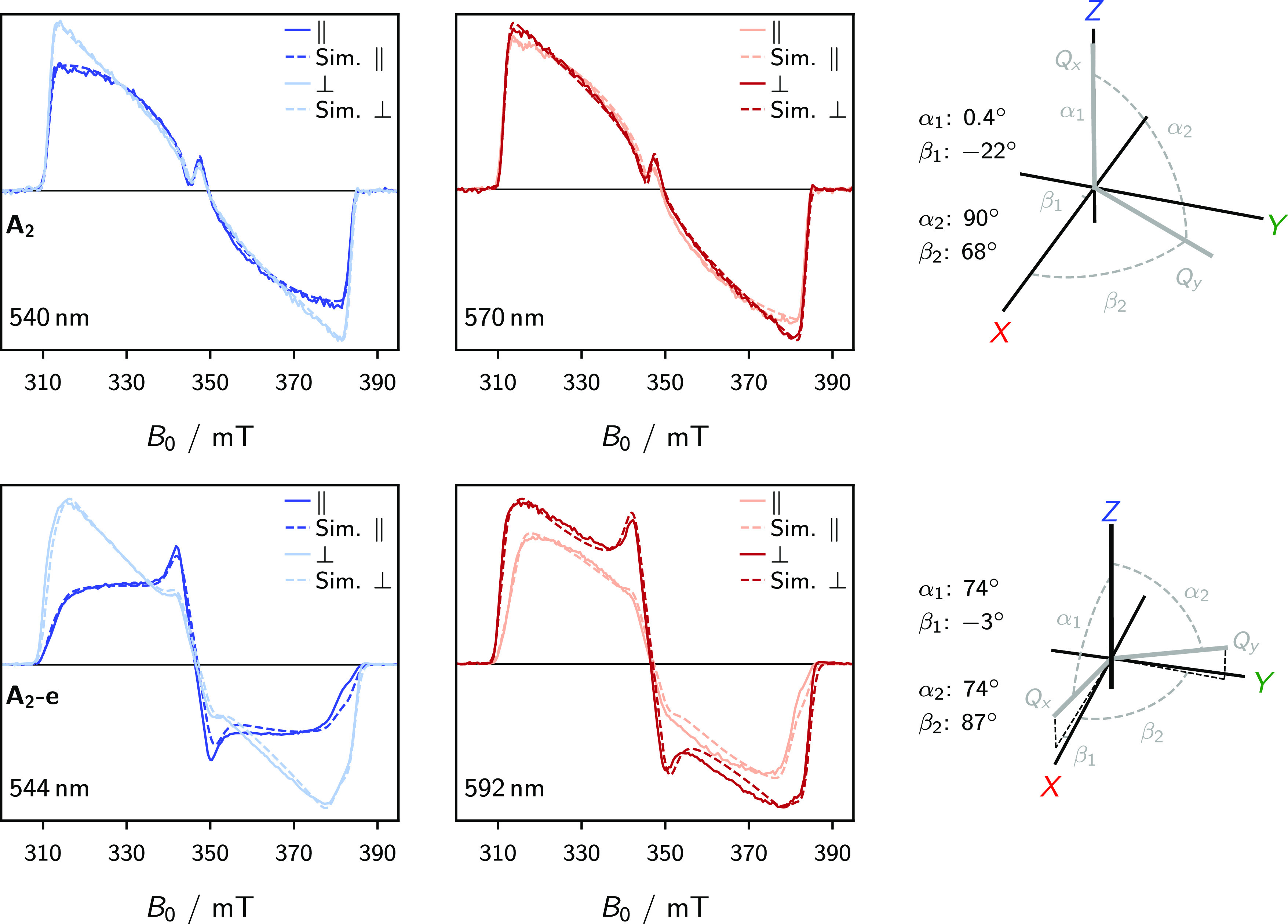
Experimental and simulated MPS spectra recorded at the X-band for **A**_**2**_ (top) and **A**_**2**_**-e** (bottom). For the MPS spectra presented, excitation was performed with linearly polarized laser light, aligned either parallel (∥) or perpendicular (⊥) to the applied magnetic field direction. The laser wavelengths specified in each panel correspond to absorptions in the optical Q-band region of these porphyrins (see SI). The α and β angles define the relative orientations of the principal axes of the ZFS tensor (*X*, *Y*, and *Z*) and the optical transition dipole moments (*Q_x_* and *Q_y_*), depicted in the diagrams adjacent to the spectra and were obtained by least-squares fitting. The EPR fitting parameters are given in Table 1. The simulations for *D*-values of opposite signs as well as a more in-depth discussion of the fitting procedures and interpretation are presented in the SI.

Figure 3 shows the results of MPS experiments performed on the **A**_**2**_ and **A**_**2**_**-e** systems, where clear intensity changes across the electron-spin polarization pattern are observed for different light polarizations. For a quantitative interpretation of these changes, the MPS spectra have been simulated by explicitly accounting for photoselection effects. The simulated spectra are also displayed in Figure 3. It is important to note that, throughout the remainder of this discussion, the following assumptions are made: (1) all porphyrin systems have two perpendicular optical transition dipole moment (TDM) vectors located within the molecular plane^51,52^ and (2) provided the two Q-bands occupy distinct regions of the absorption spectrum, the two TDMs can be excited independently by operating the laser at a suitable wavelength. The wavelengths chosen for these particular MPS measurements correspond to the Q-band region of the absorption spectrum. This region consists of two bands corresponding to the two transition moments. From the simulations and associated parameters shown in Figure 3, we conclude that the best agreement with the experiment was obtained for a negative *D*-value in the case of **A**_**2**_ and a positive *D*-value for **A**_**2**_**-e**. Further discussion of the MPS experiment, as well as additional simulations that address the uniqueness of the fitting results, are provided as part of the SI.

For **A**_**2**_**-e**, the best fit values for α and β, which define the relative orientations of the principal axes of the ZFS tensor (*X*, *Y*, and *Z*) and the optical transition dipole moments (*Q_x_* and *Q_y_*), imply that the two optical moments are approximately collinear with the *X* and *Y* ZFS axes. This proves the assumption of a positive *D*-value for **A**_**2**_**-e**, as observed previously in zinc monomer systems.^28,29^ By contrast, the angles obtained for **A**_**2**_ indicate that the ZFS *Z*-axis is in the plane of the porphyrin. In other words, the **D**-tensor is reoriented in **A**_**2**_ versus **A**_**2**_**-e**. The physical interpretation of this reorientation is both paramount and highly nontrivial: the main source of complication arises from the fact that the orthorhombicity parameter, |*E*/*D*|, is very close to 1/3. In previous investigations, which compared monomer and dimer systems, a reorientation of the **D**-tensor coincided with a shift toward a prolate spin density.^53−56^ The arguments therein proposed have been invoked to explain the small changes in the magnitudes of the *D*-value between the monomer and dimer systems despite the larger delocalization length in the dimer. In **A**_**2**_, the magnitude of the *D*-value is similar to the other systems (Table 1), despite no significant change in the delocalization length. This can only be attributed to the large |*E*/*D*| value, indicating that the spin density is far from an idealized prolate distribution and hence that the sign of *D* alone is no longer a good measure of the symmetry of the spin density. For the remaining systems, the absence of clear changes in the relative canonical intensities between excitation at different wavelengths within the Q-band region precluded further analysis of the MPS experiments (see the SI for additional data).

### Zero-Field Splitting Interaction

**Figure 4:**
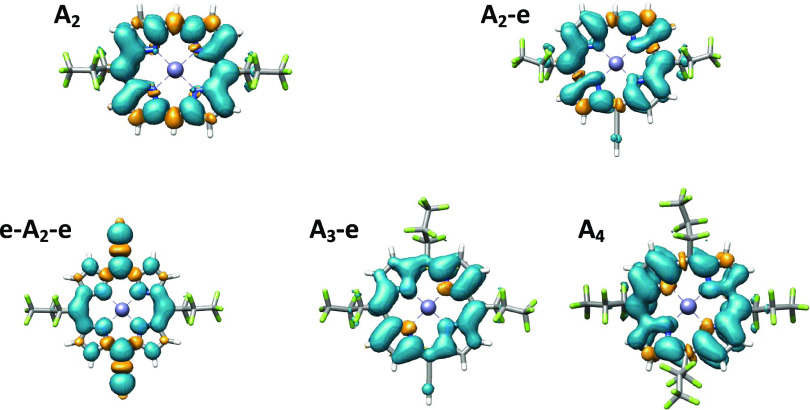
Visualization of the spin-density distributions of the photoexcited triplet states for the five perfluoroalkyl-substituted porphyrins calculated at the B3LYP/EPR-II level.

To more fully interpret the transient EPR results, quantum chemical calculations were performed using the DFT and CASSCF methods. Inspection of the optimized structures, obtained using DFT, reveals varying degrees of deviations from planarity across this series of porphyrin molecules (Figure 4). We find that **A**_**4**_ deviates most from planarity, whilst **e-A**_**2**_**-e** is relatively flat. The spin density for the flat **e-A**_**2**_**-e** system is almost identical in shape to other flat porphyrin systems where the acceptor groups are absent (e.g., replaced by aryl groups).^29^ For our remaining systems, the spin-density distribution is clearly altered from the highly symmetric one determined for **e-A**_**2**_**-e**. It seems that the extent of distortion of the spin density is, at least partially, mirrored by the extent of deviation from planarity of the porphyrin core. It is clear from eq 1 that the asymmetry parameter, η_D_ = |*E*/*D*|, is reflective of the orthorhombicity of the spin density. This could explain why **e-A**_**2**_**-e** has the flattest porphyrin core, the least distorted spin density, and the smallest η_D_ value.

Bearing this argument in mind, there is generally good agreement between the shapes of the spin densities in Figure 4 and the fine-structure parameters in Table 1. However, the failure of the DFT-calculated spin–spin contribution to the *D* parameter (Table 2) to reproduce the experimental observations needs to be addressed both in this study and in future work on similar systems. The reason for this failure in computing *D*-values with DFT can partly be attributed to the multireference character of the triplet-state wave function.^47,57−60^ For clarity, the lowest energy triplet-state wave function of porphyrin molecules is an admixture of more than one of the canonical triplet-state wave functions obtainable in the context of DFT. For this reason, the results obtained using CASSCF are in better agreement with the experimental results in both magnitude and, importantly, trend (Table 2). In these CASSCF calculations, the active space is composed of four electrons and four molecular orbitals (i.e., a CAS(4,4) calculation). The chosen active molecular orbitals are reminiscent of the Gouterman four-orbital model used to interpret the optical spectra of many porphyrin systems.^51,52^ The improvements over DFT methods, in the calculated *D*-values shown here, indicate that the CAS(4,4) wave functions seem to encapsulate the multireference character of these triplet states. However, further CASSCF investigations into these highly delocalized porphyrin triplet states are, in light of this work, imperative. Particularly, increasing the size of the active space would be desirable in promoting our understanding of the true interplay between the electronic structure and magnetic properties of porphyrins such as these, whereby the previous methodologies no longer seem sufficient. One interesting observation at this point might be that DFT and CASSCF do not agree on the sign of *D* for **A**_**2**_ (and indeed some other porphyrins). The error in the CASSCF sign of *D* for **A**_**2**_, in the context of the experimental data, is an open question, which we tentatively attribute to the complex electronic symmetry of the system. For a highly orthorhombic zero-field splitting interaction (Table 2), any small deviations from the calculated tensor may lead to an exchange of principal axis assignment, along with a concomitant change in sign for *D*.

**Table 2:** Comparison of the Experimental ZFS Parameters with Results from Quantum Chemical Calculations Using the DFT and CASSCF Methods

	experimental		DFT		CASSCF	
	*D*/MHz	η_D_	*D*/MHz	η_D_	*D*/MHz	η_D_
**A**_**2**_	–1017	0.33	–863	0.16	+939	0.29
**A**_**2**_**-e**	+1033	0.26	–824	0.18	+936	0.28
**e-A**_**2**_**-e**	+1074	0.10	+575	0.17	+976	0.29
**A**_**3**_**-e**	+1000	0.21	+594	0.18	+862	0.10
**A**_**4**_	+967	0.31	–725	0.14	+739	0.25

Therefore, both CASSCF and DFT clearly fail to predict the correct sign of *D*. Further theoretical developments are imperative if the sign of *D* from calculations is to be employed as a key predictor for the extent and symmetry of spin delocalization.

### Mims ENDOR

**Figure 5:**
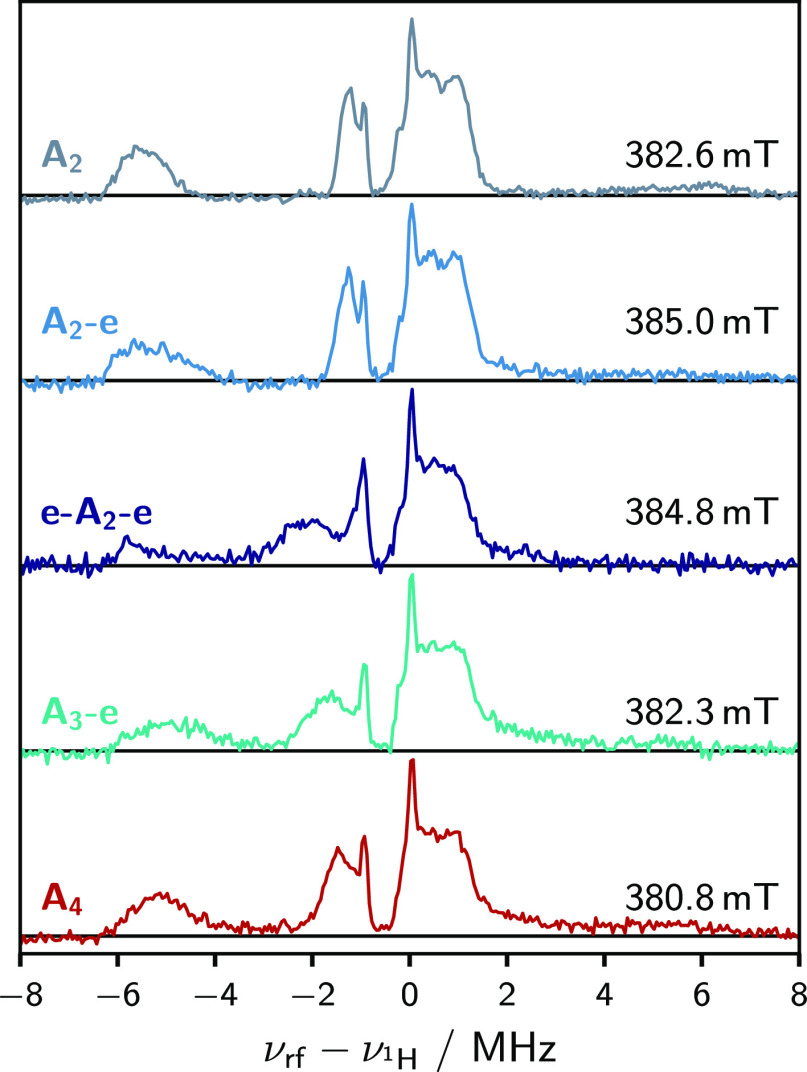
Proton Mims ENDOR spectra for all compounds recorded at the specified magnetic field position. The resonance fields correspond to both the *X* and *Z* canonical orientations of the **D**-tensor. The extent to which the *X* orientation contributes to the spectrum depends on the orthorhombicity of the **D**-tensor. The narrow and intense lines located at ≈−1 and 0 MHz, present in all spectra, correspond to the ^19^F and ^1^H Larmor frequencies, respectively.

Proton ENDOR measurements were performed on all compounds and are presented in Figure 5. The spectra were acquired at the high-field edge of the trEPR spectra. For **A**_**2**_**-e**, **e-A**_**2**_**-e**, and **A**_**3**_**-e**, this primarily corresponds to the *Z* orientation, whereas in the more orthorhombic systems, **A**_**2**_ and **A**_**4**_, the spectrum contains contributions from both the *X* and *Z* orientations. All systems display an intense narrow peak at 0 MHz and approximately −1 MHz (relative to the proton Larmor frequency, ≈14 MHz), attributed to the ^1^H and ^19^F Larmor peaks, respectively. The assignment of the remaining peaks is less clear. A Gaussian fitting of multifrequency and multifield ENDOR spectra for **A**_**2**_**-e**, shown in the SI, suggests that both ^1^H and ^19^F hyperfine couplings contribute to the observed spectrum. Further, the largest hyperfine couplings in the spectrum (>4 MHz) are primarily due to ^1^H couplings, whereas rigorous discrimination of the other peaks is less straightforward. However, importantly, across the series, the overall spectral appearance is largely invariant, with hyperfine peaks centered at approximately −5, −1.5, and 0.7 MHz. The small magnitude and similarity of the ^19^F couplings makes it difficult to assign an origin of the coupling, Fermi contact or dipolar, and prevents any interpretation as to the extent of spin delocalization.

Previously, the orientation selection exhibited in triplet ENDOR has been exploited to support conclusions of the **D**-tensor orientation in monomer and dimer porphyrin systems.^29^ For **A**_**2**_ as well as **A**_**4**_, however, the significantly orthorhombic nature of the ZFS tensor does not allow deconvolution of both *X* and *Z* components, as was exploited in our earlier work. The lack of distinguishing features in the ENDOR spectra, recorded at the *Y* orientation, renders truly quantitative interpretation of these ENDOR spectra almost impossible for these donor–acceptor systems (see the SI for further information and spectra).

However, for **A**_**2**_**-e**, **e-A**_**2**_**-e**, and **A**_**3**_**-e**, interpretation of the data is simplified by the facts that (a) only the *Z* canonical orientation contributes to the ENDOR spectra, (b) the ENDOR spectra of all three species were recorded on the high-field side of the barycenter of their time-resolved EPR spectrum, and (c) the observed hyperfine signals are consistently positioned to the left of the corresponding Larmor peak. Now, as long as we make the reasonable assumption that both the sign of the hyperfine couplings and the relative orientation between the hyperfine coupling tensors and the *Z*-axis are preserved in these systems, relative to the reference system **P1** (see the SI), the assignment of a positive *D*-value is confirmed.

In summary, whilst the large orthorhombicity in the ZFS tensor of **A**_**2**_ and **A**_**4**_ inhibits the determination of the sign of *D* from the ENDOR spectra, the possibility to isolate the *Z*-component in the ENDOR spectrum of the other chromophores further supports the assignment of a positive *D*-value.

## Conclusions

The EPR and computational results discussed here are consequential on a number of fronts, as they (1) highlight the complexities that might be encountered in interpreting EPR spectra of photogenerated triplet states, (2) provide guidance for future synthetic efforts in the field of porphyrin chemistry, and (3) demonstrate the urgency for developing more sophisticated theoretical frameworks to keep up with synthetic and experimental EPR advances. The modeling of any EPR fine-structure parameters and therefore elucidation of the spin densities in supramolecular structures is only possible if such theoretical frameworks are now evolving.

We have clearly shown that an unequivocal assignment for the ZFS *D* and *E* parameters and therefore spin-density symmetry is not always trivial, or indeed meaningful. The *D*- and *E*-values determined, particularly for the **A**_**2**_ system, have a very subtle relationship with the spin density. This work shows that when η_D_ approaches 1/3, the sign of *D* cannot meaningfully be reconciled with the shape of the spin density.

However, even in the case of η_D_ ≈ 1/3, the sign of *D* in itself is neither meaningless nor inconsequential. The sign of *D* still determines the energetics of the triplet spin sublevels, their populations, and therefore the intersystem crossing characteristics. Hence, although the sign of *D* cannot be used as a predictor of either the extent or symmetry of the spin-density delocalization in **A**_**2**_, with η_D_ = 0.33, its determination from MPS allows us to conclude that the SOC is driving the triplet sublevel populations into the out-of-plane sublevel, analogous to the other porphyrins in this series of monomers. For **A**_**2**_, however, this is now the *X*-axis rather than the *Z*-axis.

From the synthetic point of view, it is clear that if EPR is to be used to investigate the spin density in acceptor porphyrins, then the substitution pattern and orthorhombicity of the molecules have to be carefully considered.

The importance of photogenerated triplet states in devices designed for applications in solar energy conversion, molecular electronics, and spintronics is ever increasing.^2,61−63^ The development of robust computational methods that allow the characterization of the ZFS tensor, independent of its symmetry, is therefore crucial. We believe that this series of porphyrin structures with their complex zero-field splitting symmetry offer an ideal testing ground for the benchmarking of such new methodology.

## Supporting Information Available

Additional magnetophotoselection data and simulations, additional ENDOR data and decomposition, and DFT geometries (PDF)
