# Supplementary material for: EPR of Photoexcited Triplet-State Acceptor Porphyrins
Source: J Phys Chem C Nanomater Interfaces. 2021 May 19;125(21):11782–90. doi: 10.1021/acs.jpcc.1c03278 (PMC8279703; doi:10.1021/acs.jpcc.1c03278)
Supplement: Supplementary file 1 — jp1c03278_si_001.pdf [file jp1c03278_si_001.pdf]

Supporting Information for

EPR of Photoexcited Triplet State Acceptor Porphyrins

Ashley J. Redman,[†,§] Gabriel Moise,[†,§] Sabine Richert,[‡] Erin J. Viere,[¶] William K. Myers,[†] Michael J. Therien,[¶] and Christiane R. Timmel[†,*]

†*Centre for Advanced Electron Spin Resonance (CÆSR), University of Oxford, South Parks Road, Oxford OX1 3QR, United Kingdom*

‡*Institute of Physical Chemistry, University of Freiburg, Albertstraße 21, 79104 Freiburg, Germany*

¶*Department of Chemistry, Duke University, French Family Science Center, 124 Science Drive, Durham, North Carolina 27708, United States*

§These authors contributed equally.

∗ email: christiane.timmel@chem.ox.ac.uk

Contents

S1 Benchmarks **S1**

S2 Electronic Absorption Spectroscopy **S2**

S3 Spin Polarisation and Intersystem Crossing **S2**

S4 Magnetophotoselection **S3**
S4.1 Additional Simulations S6
S4.2 Additional Data S12

S5 ENDOR **S17**
S5.1 **A_2-e** Nuclide Assignment S17

S6 DFT Calculations—Structures and Spin Densities **S18**

S1 Benchmarks

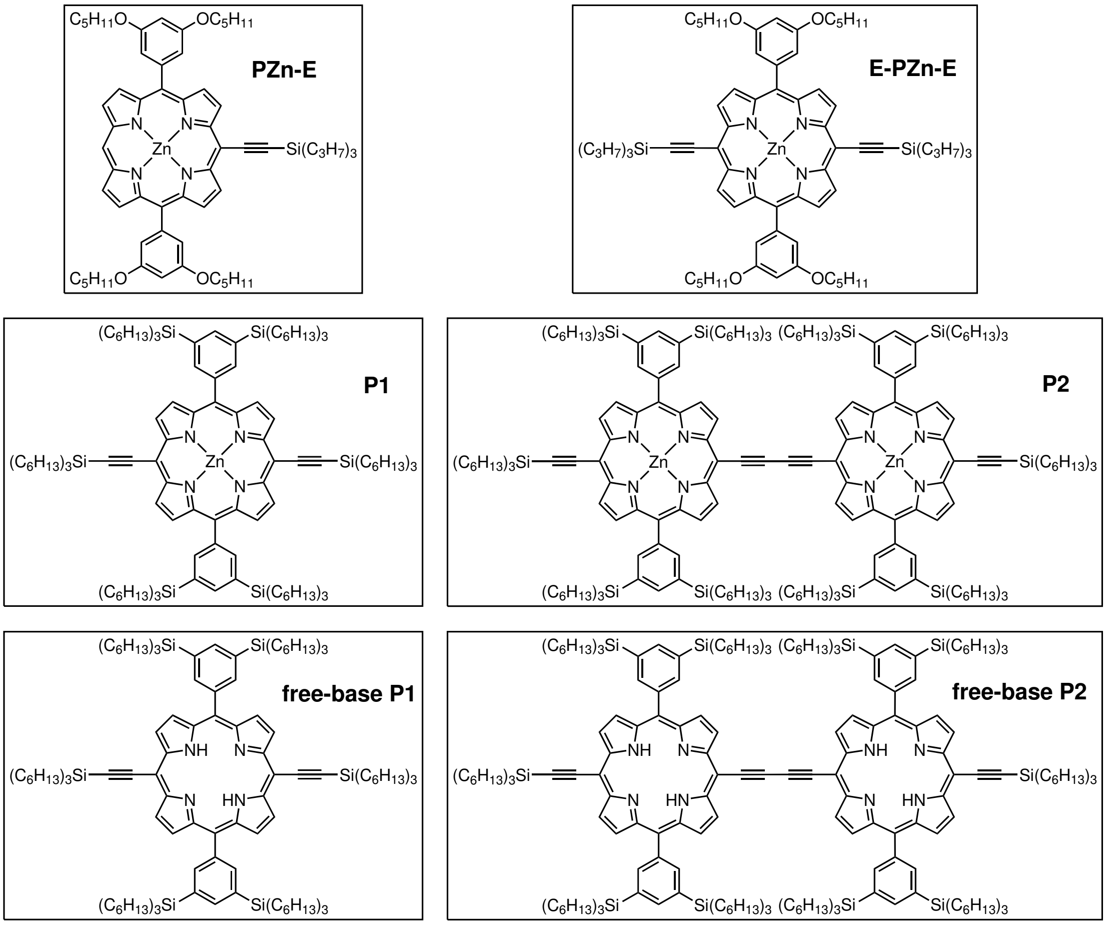

Figure S1: Chemical structures for the benchmark systems from literature cited in the main text. Refer to **Table S1** for additional information.

Table S1: ZFS parameters and triplet sublevel populations for the benchmark systems taken from literature cited in the main text, alongside the systems considered in this work. For the compounds considered in previous work, the naming conventions introduced in the primary literature has been preserved. † The reported triplet sublevel populations taken from Ref. [10] have been renormalised to the conventions used in this work to enhance the ease of comparison, this does not affect the interpretation of the data. ‡ The simulation parameters for **A₂** with $D > 0$ have been included for comparison.

System	D / MHz	E / MHz	$\|E\|/\|D\|$	$P_{\{X,Y,Z\}}^{\dagger}$	Reference
free-base P1	1024	−144	0.141	0.47, 0.53, 0.00	[9]
free-base P2	−1053	311	0.295	0.00, 0.29, 0.71	[9]
P1	898	−161	0.179	0.05, 0.00, 0.95	[9]
P2	−1117	284	0.254	0.88, 0.00, 0.12	[9]
PZn-E	962	−167	0.174	0.11, 0.00, 0.89	[10]
E-PZn-E	919	−162	0.176	0.00, 0.02, 0.98	[10]
**A_2**	−1017	333	0.327	0.81, 0.00, 0.19	This work
**A_2-e**‡	1017	−333	0.327	0.00, 0.57, 0.43	This work
**A_2-e**	1033	−272	0.263	0.10, 0.00, 0.90	This work
**e-A_2-e**	1074	−105	0.098	0.00, 0.10, 0.90	This work
**A_3-e**	1000	−214	0.214	0.05, 0.00, 0.95	This work
**A_4**	967	−300	0.310	0.22, 0.00, 0.78	This work

S2 Electronic Absorption Spectroscopy

UV-vis measurements of all compounds were performed at room temperature in 2-MeTHF solution, **Figure S2**. The spectra were recorded using a Shimadzu UV-1601 UV-vis spectrophotometer.

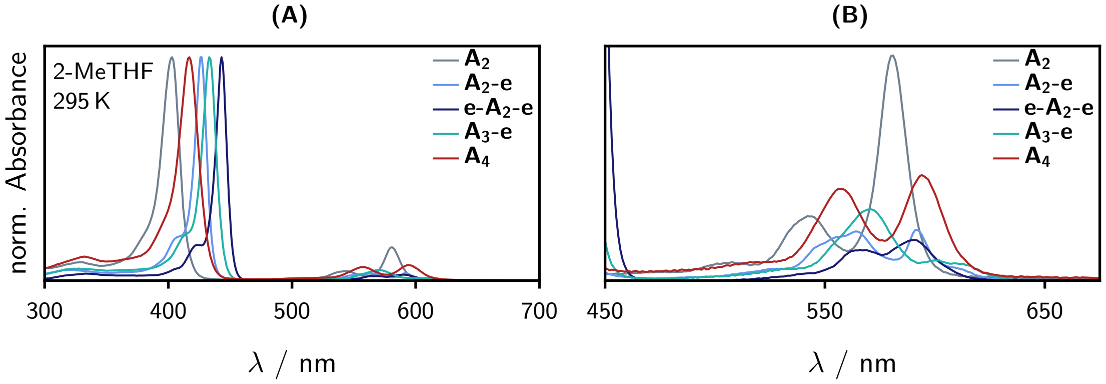

Figure S2: Room temperature electronic absorption spectra for all compounds. **(A)** displays the spectra recorded from 300 nm to 700 nm, whereas **(B)** displays an enhancement of the porphyrin Q-band region.

S3 Spin Polarisation and Intersystem Crossing

An excited singlet state is generated upon application of a laser pulse. Intersystem crossing between the excited singlet and triplet states selectively populates the triplet sublevels generating the spin polarised triplet state. At zero magnetic field, the triplet state has three sublevels (denoted $T_{X,Y,Z}$) which are split by the zero-field splitting interaction, parametrised by the D- and E-values. Where the $_{X,Y,Z}$ are related to the principle axes of the $\boldsymbol{D}$-tensor. The sublevel populations reported correspond to the zero-field

eigenstates, namely $T_{X,Y,Z}$, which can be related to the high field populations by linear combinations, *viz*

$$P_0 = P_Z \qquad P_{\pm1} = \frac{1}{2}(P_X + P_Y) \qquad \text{for } \boldsymbol{B} \parallel Z$$

The selective population in zinc porphyrins is dominated by a direct spin–orbit coupling term of the form $\langle T_1|\mathcal{H}_{SO}|S_1\rangle$, arising from a one center term on the zinc atom due to a mixing of the d-orbitals with the porphyrin π system. This effect occurs for the out-of-plane sublevel, which, for a positive D-value, corresponds to T_Z.

S4 Magnetophotoselection

Numerical simulations of a typical trEPR experiment cannot reveal the sign of the D-value. Identical simulations can be produced using opposite signs for D through a re-distribution of the triplet sublevel populations. This is demonstrated in **Figure S3**, where A and D and B and C display equivalent spectra.

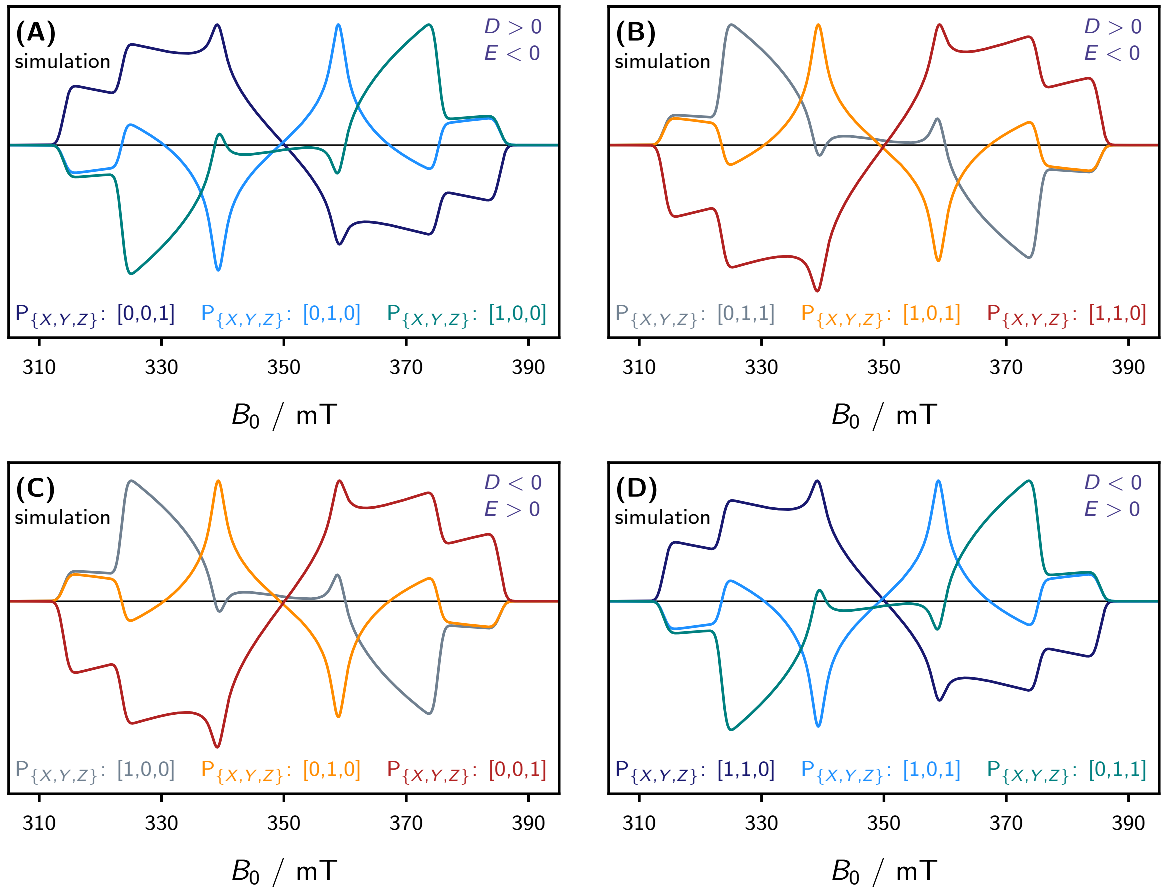

Figure S3: The polarisation pattern observed in a trEPR spectrum depends on both the relative sublevel populations and the sign of the ZFS D-parameter. The changes between **(A)** and **(B)** arise from the changes in populations, whereas the changes between **(A)** and **(C)** are due to the change in sign of D. By comparing **(A)** and **(D)** it is clear that identical spectra can be obtained in this case through a redistribution of the populations for the simulation with $D < 0$. Isolating the parameter(s) responsible is not generally possible from a typical trEPR experiment and additional information is required.

The magnetophotoselection experiment is an extension of the trEPR technique which can provide experimental evidence for the sign of the D-value.

The probability, κ, of the absorption of light is given by

$$\kappa = (\boldsymbol{\mu} \cdot \boldsymbol{\nu})^2$$

where $\boldsymbol{\mu}$ is the optical transition dipole moment and $\boldsymbol{\nu}$ is the electric field vector of the light. The orientation of $\boldsymbol{\mu}$ and $\boldsymbol{\nu}$ can be specified relative to the ZFS tensor using spherical angles, as demonstrated in **Figure S4**. The α, β angles define the direction of the optical transition moment with respect to the orientation of the $\boldsymbol{D}$-tensor. For porphyrin structures, the Q-band region corresponds to excitation along one of two orthogonal moments in the plane of the ring, namely, Q_x and Q_y. The θ and ϕ angles specify the orientation of the applied magnetic field relative to the ZFS tensor frame of the molecule.

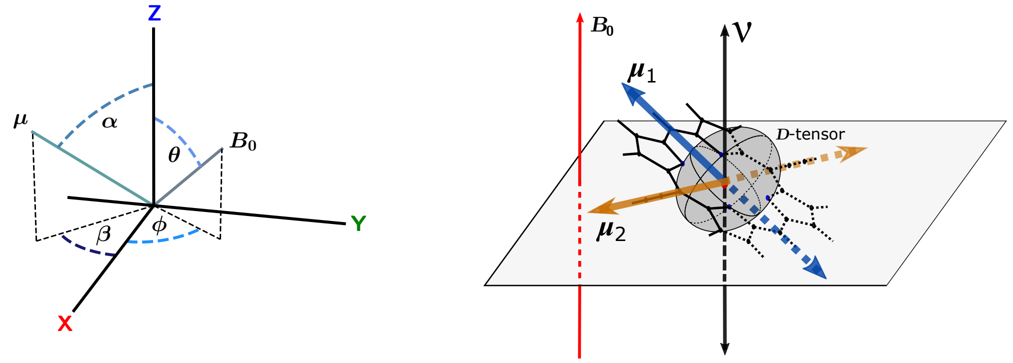

Figure S4: Position of the optical transition moment $\boldsymbol{\mu}$ and the field vector $\boldsymbol{B}_0$ relative to the $\boldsymbol{D}$-tensor frame (X, Y, Z) (*left*). Visualisation of the $\boldsymbol{D}$-tensor projected onto a porphyrin plane, indicating relative orientations of the field and optical transition moments $\boldsymbol{\mu}_{1,2}$ as well as $\boldsymbol{\nu}$ (*right*).

During a magnetophotoselection experiment, the laser light is polarised either parallel (∥) or perpendicular (⊥) relative to the field direction.
The $(\boldsymbol{\mu} \cdot \boldsymbol{\nu})^2$ term provides a weighting factor for a specific $(\alpha, \beta, \theta, \phi)$ orientation. General equations for a defined polarisation have previously been derived by several authors [7]:
1. For depolarised light,

$$\begin{aligned}(\boldsymbol{\mu} \cdot \boldsymbol{\nu})^2_\circ = {} & [11 + \cos 2\theta \\ & + 2\, \cos\left(2\left(\beta - \phi\right)\right) \sin^2\theta\left(1 - \cos 2\alpha\right) \\ & + \cos 2\alpha \left(1 + 3\, \cos 2\theta\right) \\ & + 4\, \cos\left(\beta - \phi\right) \sin 2\alpha\, \sin 2\theta]\, /32\end{aligned}$$

2. For the light electric field vector aligned *parallel* to the field, $B_0 \parallel \nu$,

$$\begin{aligned}(\boldsymbol{\mu} \cdot \boldsymbol{\nu})^2_\parallel = & \Bigg[2\, \cos\left(\alpha - \theta\right) + \cos\left(\alpha + \beta - \phi - \theta\right) \\ & + \cos\left(\alpha - \beta + \phi - \theta\right) \\ & + 4\, \cos\left(\alpha + \theta\right) \sin^2 \frac{\left(\beta - \phi\right)}{2}\Bigg]^2 /16\end{aligned}$$

3. For the light electric field vector aligned *perpendicular* to the field, $B_0 \perp \nu$,

$$\begin{aligned}(\boldsymbol{\mu}\cdot\boldsymbol{\nu})^2_\perp = &[(6 - 2\ \cos 2\,(\beta-\phi) + \cos 2\,(\beta-\phi-\theta)\\ &+ 2\ \cos 2\theta + \cos 2\,(\beta-\phi+\theta))\sin^2\alpha\\ &+ 8\ \cos^2\alpha\ \sin^2\theta\\ &- 4\ \cos(\beta-\phi)\sin 2\alpha\ \sin 2\theta]\,/16\end{aligned}$$

A fitting of the simulation to the data can then be performed in a least-squares sense by optimising the input parameters.
The MPS simulated spectra presented are an admixture of contributions arising from excitation with polarised light and depolarised light. The requirement for this can be explained by considering the behaviour of κ in the special case where the Q-moment is along the canonical orientations of the ZFS-tensor. The expressions for κ can then be reduced to

$$\begin{array}{ll} & (\boldsymbol{\mu}\cdot\boldsymbol{\nu})^2_\parallel\\ x: & \sin^2\theta\ \cos^2\phi\\ y: & \sin^2\theta\ \cos^2\phi\\ z: & \cos^2\theta\end{array} \tag{1}$$

$$\begin{array}{ll} & (\boldsymbol{\mu}\cdot\boldsymbol{\nu})^2_\perp\\ x: & \frac{1}{2}\left(\cos^2\phi\ \cos^2\theta + \sin^2\phi\right)\\ y: & \frac{1}{2}\left(\sin^2\phi\ \cos^2\theta + \cos^2\phi\right)\\ z: & \frac{1}{2}\ \sin^2\theta\end{array} \tag{2}$$

Inspection of **Equations (1) and (2)** indicates when the two Q-moments are collinear with X and Y, for $B_0 \parallel Z, \kappa = 0$ and this orientation will not contribute to the final spectrum. Whereas for $B_0 \parallel X, \kappa = 0$ for *parallel* excitation along one Q-moment and *perpendicular* excitation along the other Q-moment and *vice versa* for $B_0 \parallel Y$. Together these predict pronounced spectrum intensity changes for the canonical orientations for certain combinations of laser polarisation and Q-moment excitation. This predicted complete attenuation is not realised here or in previously reported MPS investigations [8].

The magnetophotoselection spectra were simulated using a in-house routine. The peak-list was computed via full matrix diagonalisation using the EasySpin `resfields` function and an additional orientational weighting factor was computed as described abovesec:Magnetophotoselection. The spectral construction was based on methods previously reported [2, 3]. Least-squares fitting was used to obtain the reported α and β angles. During the fitting, several constraints were imposed on the parameters:

- The two optical transition moments were taken as being orthogonal.
- For a given molecule, the fitting imposed the same orientation of the transition moments for all the polarizations and wavelengths and varied the contribution of each in the simulation.
- Given the lack of ideal photoselection conditions, *vide infra*, a contribution from excitation with non-polarised light was included. The relative contribution of which was held constant over a given fitting.

S4.1 Additional Simulations

The magnetophotoselection simulations reported in the main text were performed according to simulation case D, with $D > 0$ for **A$_2$-e** and $D < 0$ for **A$_2$**, *vide infra*.
To support the tentative assignments for the sign of the D-values in **A$_2$** and **A$_2$-e**, additional simulations were performed considering a change in sign and a partial break down of the initial assumptions.

Simulation cases

- **Case A:** The zero-field splitting tensor principal axes are orientated such that one axis is fixed perpendicular to the previously defined porphyrin plane (for $D > 0$ this corresponds to the Z axis and for $D < 0$ this corresponds to the X axis). The remaining two axes are located within the porphyrin plane. The two optical transition moments, Q_x and Q_y, are associated with distinct bands in the UV–vis spectrum. During the experiment, when the laser is operated at a given wavelength, the ensemble is excited along $Q_x \veebar Q_y$.
- **Case B:** The zero-field splitting tensor principal axes are orientated such that one axis is fixed perpendicular to the previously defined porphyrin plane (for $D > 0$ this corresponds to the Z axis and for $D < 0$ this corresponds to the X axis). The remaining two axes are located within the porphyrin plane. The two optical transition moments, Q_x and Q_y, are not necessarily associated with distinct bands in the UV–vis spectrum. During the experiment, when the laser is operated at a given wavelength, the ensemble is excited along $Q_x \vee Q_y$.
- **Case C:** The zero-field splitting tensor principal axes were not assumed to have a known orientation. However, given the polarisation indices, the transition moments are located within 35° of a principal axis. The two optical transition moments, Q_x and Q_y, are associate with distinct bands in the UV-vis spectrum. During the experiment, when the laser is operated at a given wavelength, the ensemble is excited along $Q_x \veebar Q_y$.
- **Case D:** The zero-field splitting tensor principal axes were not assumed to have a known orientation. However, given the polarisation indices, the transition moments are located within 35° of a principal axis. The two optical transition moments, Q_x and Q_y, are not necessarily associated with distinct bands in the UV-vis spectrum. During the experiment, when the laser is operated at a given wavelength, the ensemble is excited along $Q_x \vee Q_y$.

For **A$_2$-e**, the best agreement with the experimental data is obtained with a positive D-value and simulation case D.

- For a given simulation case, a positive D-value always produces a better fit as determined from the RMSD values.
- Visual inspection of the simulations obtained with a negative D-value indicates incorrect relative intensities at the turning points of the spectra.
- For a positive D-value, α does not deviate far from $\pi/2$ rad, consistent with the conventions used to define a positive D-value.
- A significant contribution of polarised light (rather than powder contribution) is required for the simulations with a positive D-value and changing the wavelength occurs with a concomitant change in contribution for the two transition moments, as should be expected.

For **A$_2$**, simulations with a negative D-value lead to a subtly better fit.

- For a given simulation case, a negative D-value always produces a better fit.
- Visual inspection of both the outer and inner turning points reveals that only the negative D simulation is able to reproduce the relative intensities.
- For simulations with $D < 0$, α does not deviate far from 0 rad.
- The simulations with $D > 0$ contains unrealistic parameters for both the powder contribution and the α angle, given the polarised light used to excited the sample and the conventions used to define the sign of the D-value.

A_2-e: positive D-value

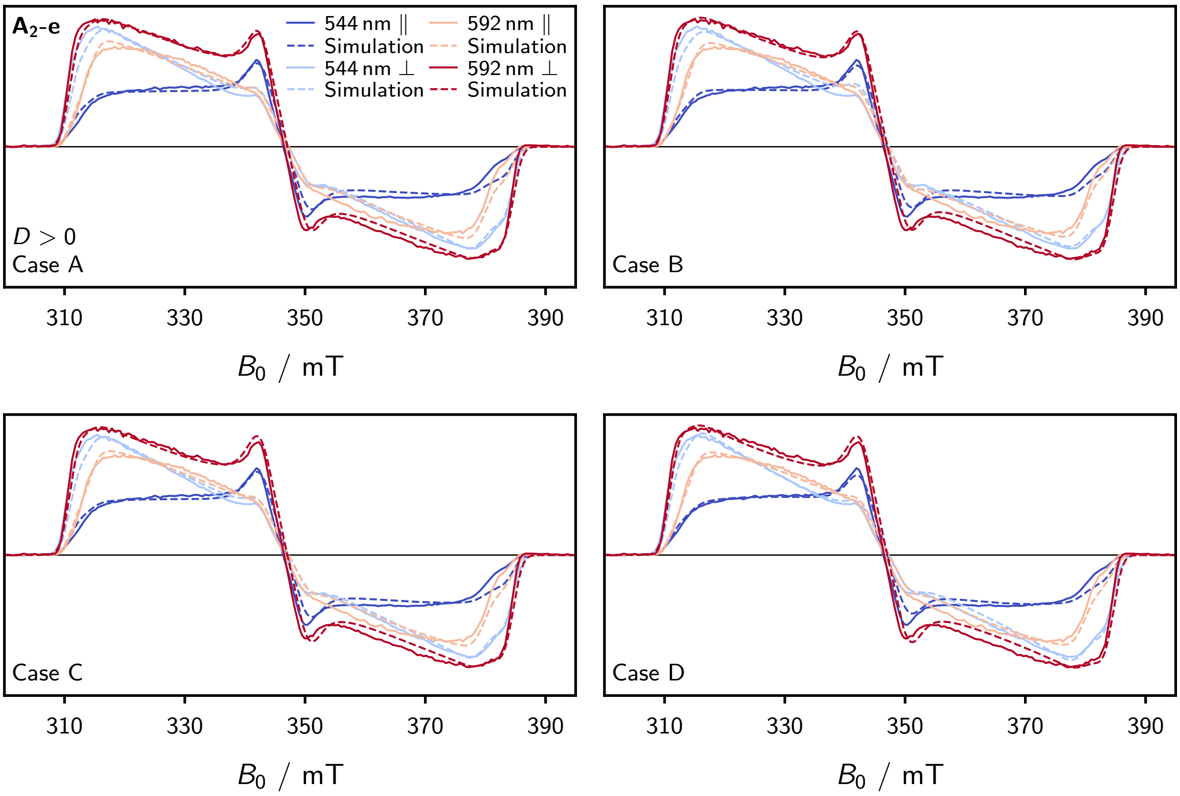

Figure S5: Experimental and simulated MPS spectra for **A_2-e** with $D > 0$. For the simulations, EPR parameters are reported in the main text and additional parameters for the MPS experiment are declared in **Table S2**. For a discussion of the simulation method see the SI text.

Table S2: Additional simulation parameters for the MPS experiment for **A_2-e** with $D > 0$.

Parameter		Simulation case A	B	C	D
α/rad		$\pi/2$ (fixed)	$\pi/2$ (fixed)	1.44317	1.29997
β/rad		-0.00798518	0.0532891	0.00325223	-0.0600764
$\lambda_{544\ \mathrm{nm}}$	Powder	0.5146	0.4273	0.4761	0.3014
	$Q_x\ [\alpha, \beta]$	–	0.1019	–	0.0899
	$Q_y\ [\alpha, \beta + \frac{\pi}{2}]$	0.4854	0.4708	0.5239	0.6088
$\lambda_{592\ \mathrm{nm}}$	Powder	0.5143	0.4174	0.4755	0.2825
	$Q_x\ [\alpha, \beta]$	0.4857	0.5514	0.5245	0.6073
	$Q_y\ [\alpha, \beta + \frac{\pi}{2}]$	–	0.0312	–	0.1102
RMSD		0.0600201	0.0579145	0.0582108	0.0521521

A_2-e: negative D-value

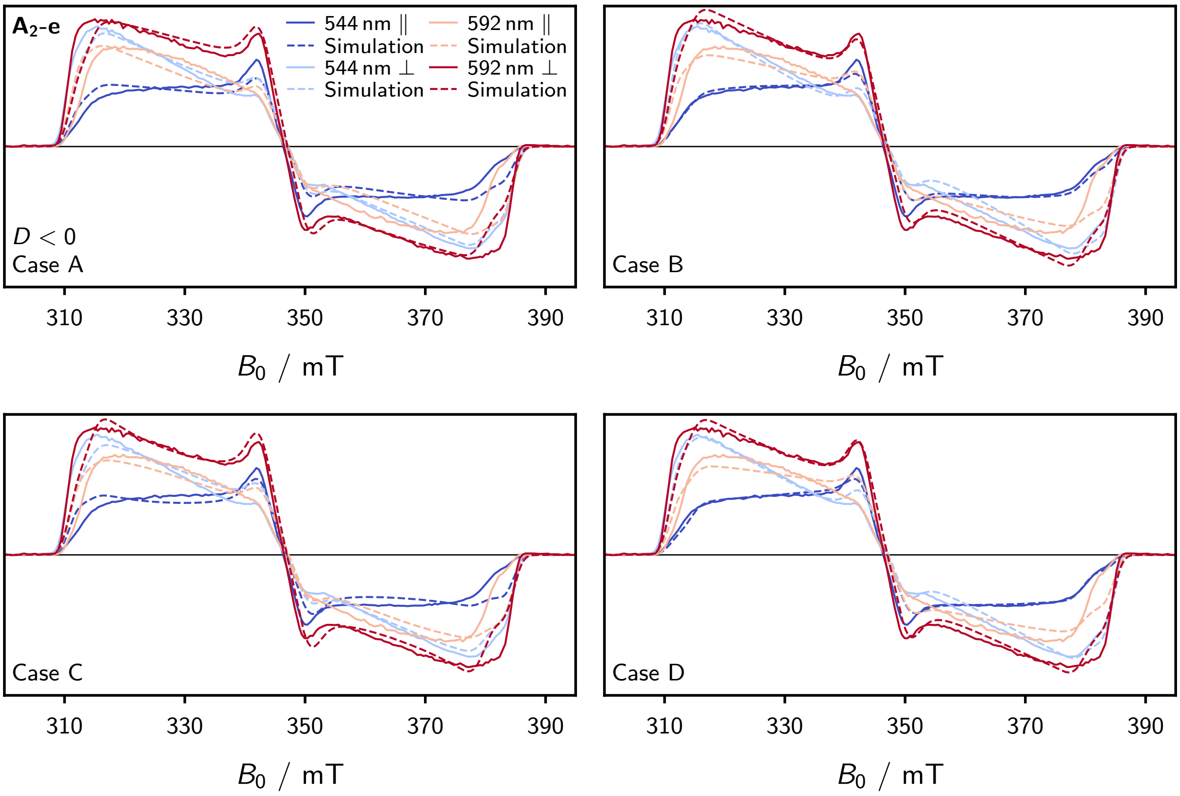

Figure S6: Experimental and simulated MPS spectra for **A_2-e** with $D < 0$. For the simulations, EPR parameters are reported in the main text (except the ZFS D and E parameters included a sign inversion and $P_{X,Y,Z} = [0.47, 0.53, 0]$) and additional parameters for the MPS experiment are declared in **Table S3**. For a discussion of the simulation method see the SI text.

Table S3: Additional simulation parameters for the MPS experiment for **A_2-e** with $D < 0$.

Parameter		Simulation case A	B	C	D
α/rad		0 (fixed)	0 (fixed)	0.610865	0.279826
β/rad		1.41247	0.919388	1.19178	0.778343
$\lambda_{544\,nm}$	Powder	0.7081	0.4123	0.5273	0.2792
	Q_x $[\alpha, \beta]$	–	0	–	0
	Q_y $[\alpha + \frac{\pi}{2}, \beta + \frac{\pi}{2}]$	0.2919	0.5877	0.4727	0.7208
$\lambda_{592\,nm}$	Powder	0.6854	0.3537	0.5088	0.2296
	Q_x $[\alpha, \beta]$	0.3146	0.3204	0.4912	0.3442
	Q_y $[\alpha + \frac{\pi}{2}, \beta + \frac{\pi}{2}]$	–	0.3259	–	0.4262
RMSD		0.104509	0.0798456	0.095869	0.0717651

A_2: positive D-value

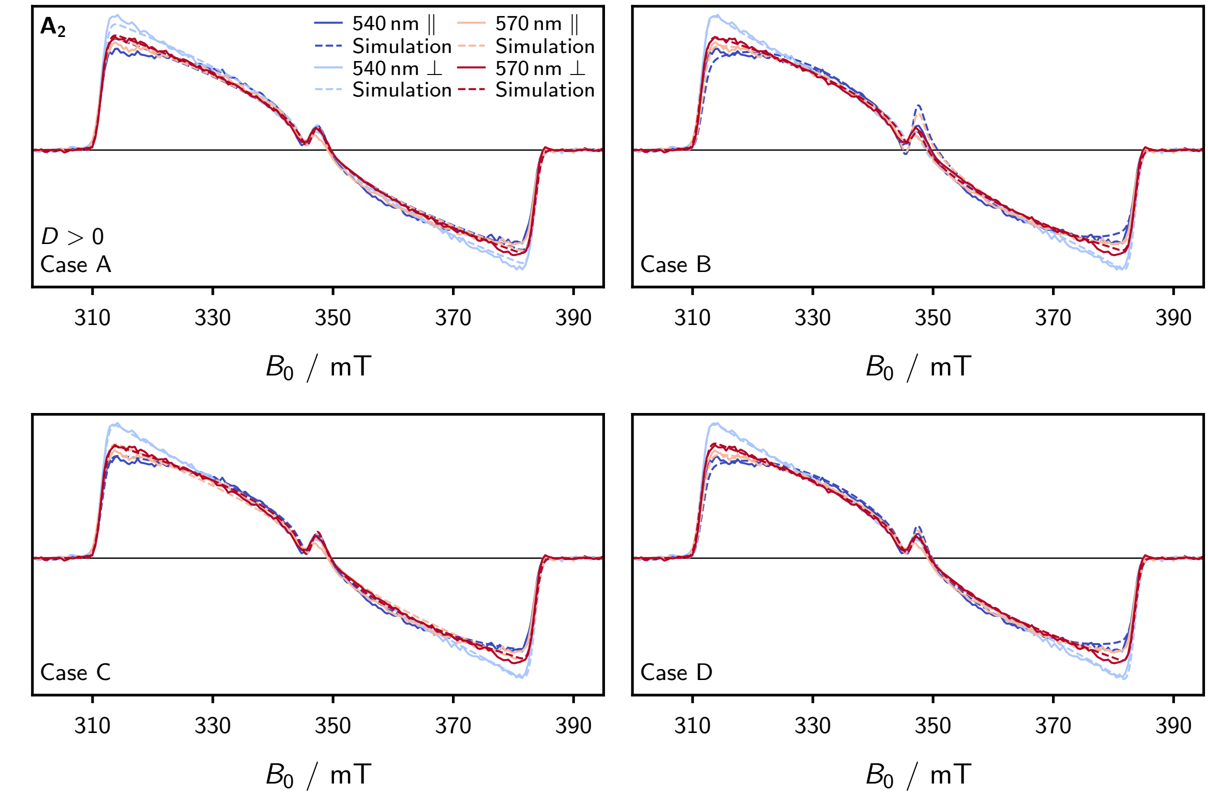

Figure S7: Experimental and simulated MPS spectra for $\mathbf{A_2}$ with $D > 0$. For the simulations, EPR parameters are reported in the main text (except the ZFS D and E parameters included a sign inversion and $P_{X,Y,Z} = [0, 0.57, 0.43]$) and additional parameters for the MPS experiment are declared in **Table S4**. For a discussion of the simulation method see the SI text.

Table S4: Additional simulation parameters for the MPS experiment for $\mathbf{A_2}$ with $D > 0$.

	Parameter	Simulation case A	B	C	D
	α/rad	$\pi/2$ (fixed)	$\pi/2$ (fixed)	0.959931	1.36675
	β/rad	-0.196627	-0.431679	-0.296421	-0.40477
$\lambda_{540\,\mathrm{nm}}$	Powder	1	0.2453	0.8243	0.3411
$\lambda_{540\,\mathrm{nm}}$	Q_x $[\alpha, \beta]$	–	0.1396	–	0.1763
$\lambda_{540\,\mathrm{nm}}$	Q_y $[\alpha, \beta + \frac{\pi}{2}]$	0	0.6151	0.1757	0.4826
$\lambda_{570\,\mathrm{nm}}$	Powder	1	0.24389	0.8257	0.3355
$\lambda_{570\,\mathrm{nm}}$	Q_x $[\alpha, \beta]$	0	0.3138	0.1743	0.3054
$\lambda_{570\,\mathrm{nm}}$	Q_y $[\alpha, \beta + \frac{\pi}{2}]$	–	0.4473	–	0.3591
	RMSD	0.0544224	0.0579145	0.582108	0.0521521

A_2: negative D-value

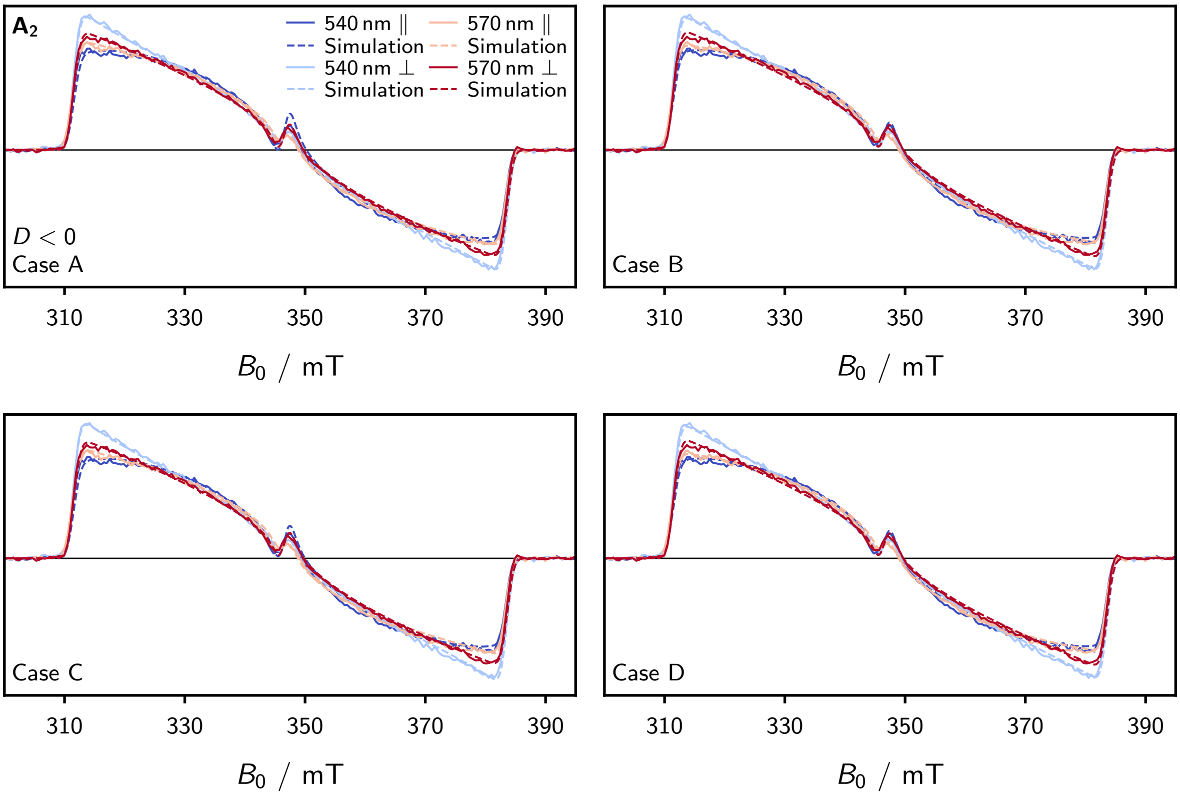

Figure S8: Experimental and simulated MPS spectra for $\mathbf{A_2}$ with $D < 0$. For the simulations, EPR parameters are reported in the main text and additional parameters for the MPS experiment are declared in S5. For a discussion of the simulation method see the SI text.

Table S5: Additional simulation parameters for the MPS experiment for $\mathbf{A_2}$ with $D < 0$.

	Parameter	Simulation case A	B	C	D
	α/rad	0 (fixed)	0 (fixed)	-0.10018	0.00703567
	β/rad	-0.387007	-0.388446	-0.420555	-0.385324
$\lambda_{540\,nm}$	Powder	0.6787	0.5882	0.6794	0.5865
$\lambda_{540\,nm}$	Q_x $[\alpha, \beta]$	–	0.1822	–	0.1886
$\lambda_{540\,nm}$	Q_y $[\alpha + \frac{\pi}{2}, \beta + \frac{\pi}{2}]$	0.3213	0.2297	0.3206	0.2270
$\lambda_{570\,nm}$	Powder	0.6799	0.5888	0.6811	0.5871
$\lambda_{570\,nm}$	Q_x $[\alpha, \beta]$	0.3201	0.3619	0.3189	0.3633
$\lambda_{570\,nm}$	Q_y $[\alpha + \frac{\pi}{2}, \beta + \frac{\pi}{2}]$	–	0.0492	–	0.0496
	RMSD	0.0368835	0.034294	0.0360747	0.0342908

S4.2 Additional Data

The MPS spectra presented in **Figures S9 to S12** show that no clear magnetophotoselection effect as a function of excitation wavelength was observed for several samples.

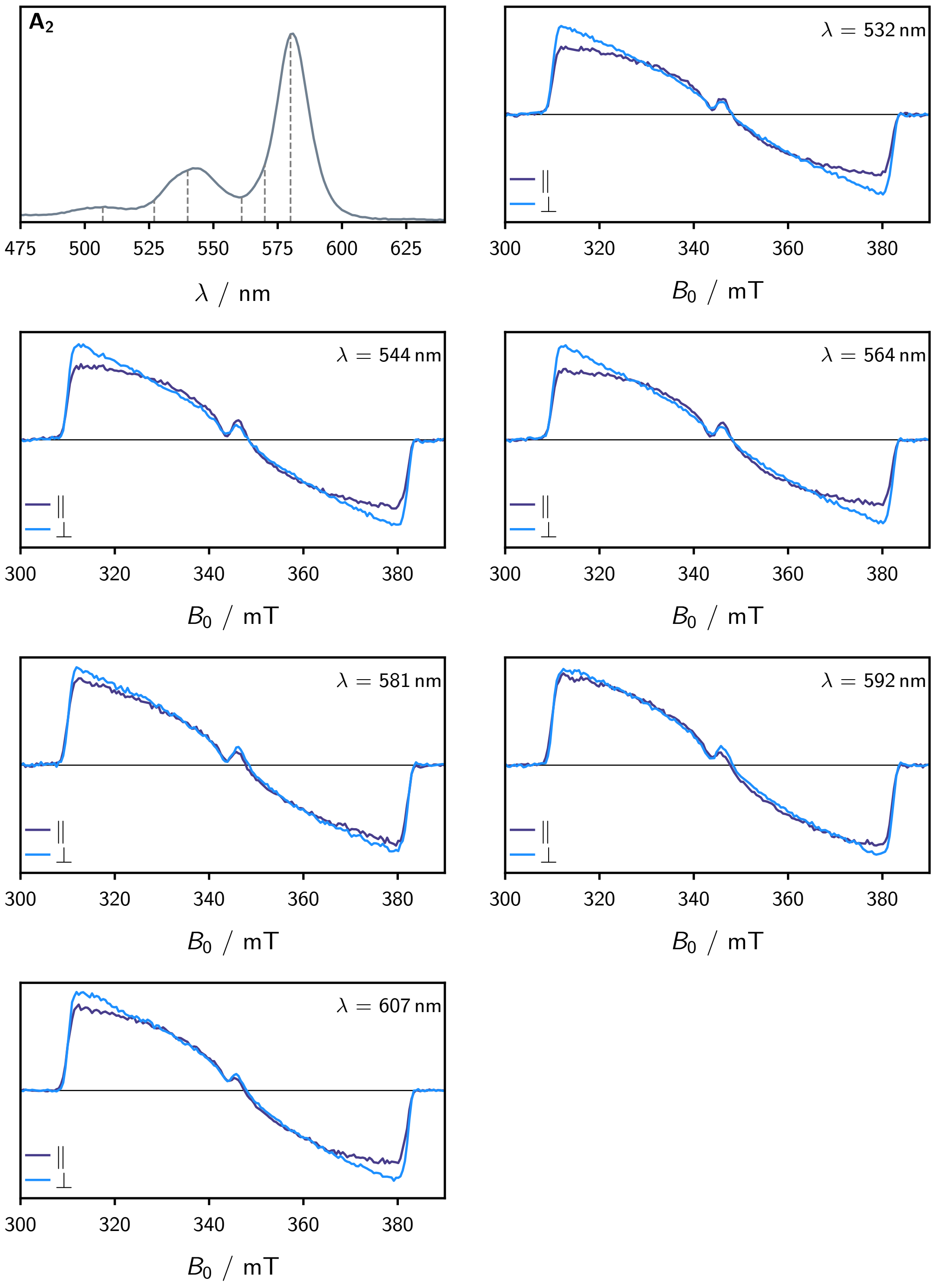

Figure S9: Electronic absorption spectrum, indicating the wavelengths chosen for MPS measurements on $\mathbf{A_2}$, alongside the MPS spectra recorded with the light polarised parallel and perpendicular to the applied field.

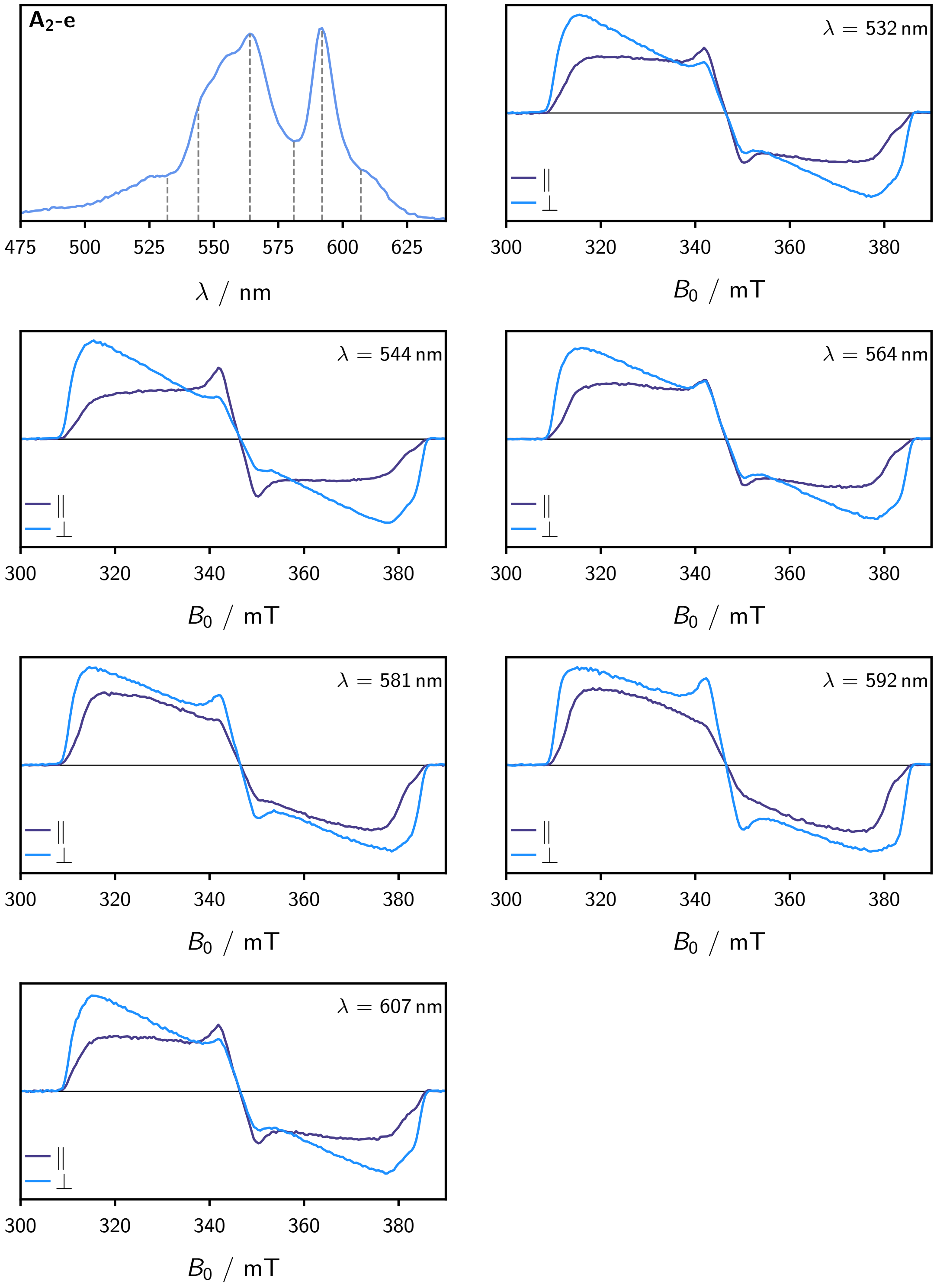

Figure S10: Electronic absorption spectrum, indicating the wavelengths chosen for MPS measurements on **A$_2$-e**, alongside the MPS spectra recorded with the light polarised parallel and perpendicular to the applied field.

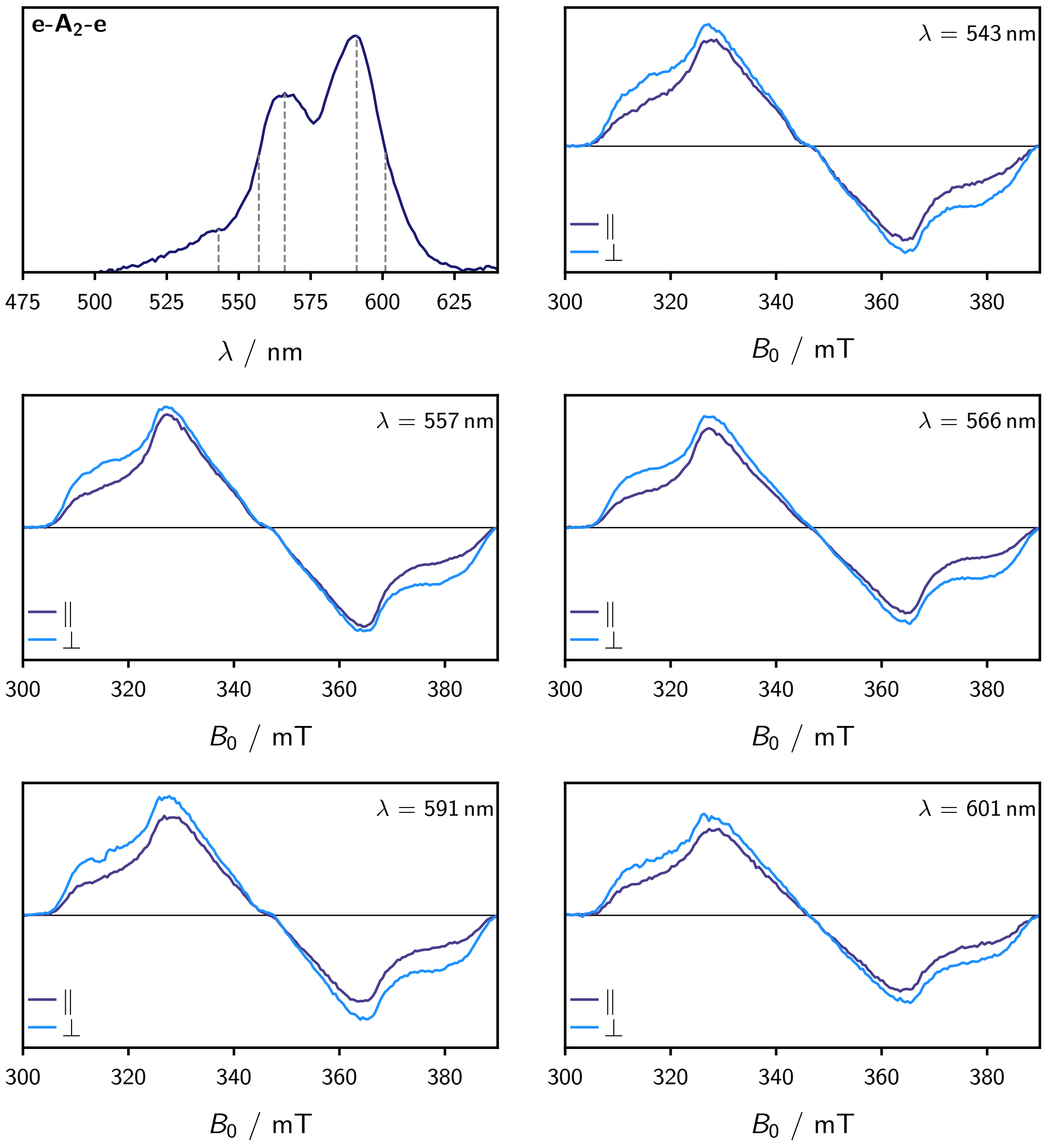

Figure S11: Electronic absorption spectrum, indicating the wavelengths chosen for MPS measurements on **e-A$_2$-e**, alongside the MPS spectra recorded with the light polarised parallel and perpendicular to the applied field.

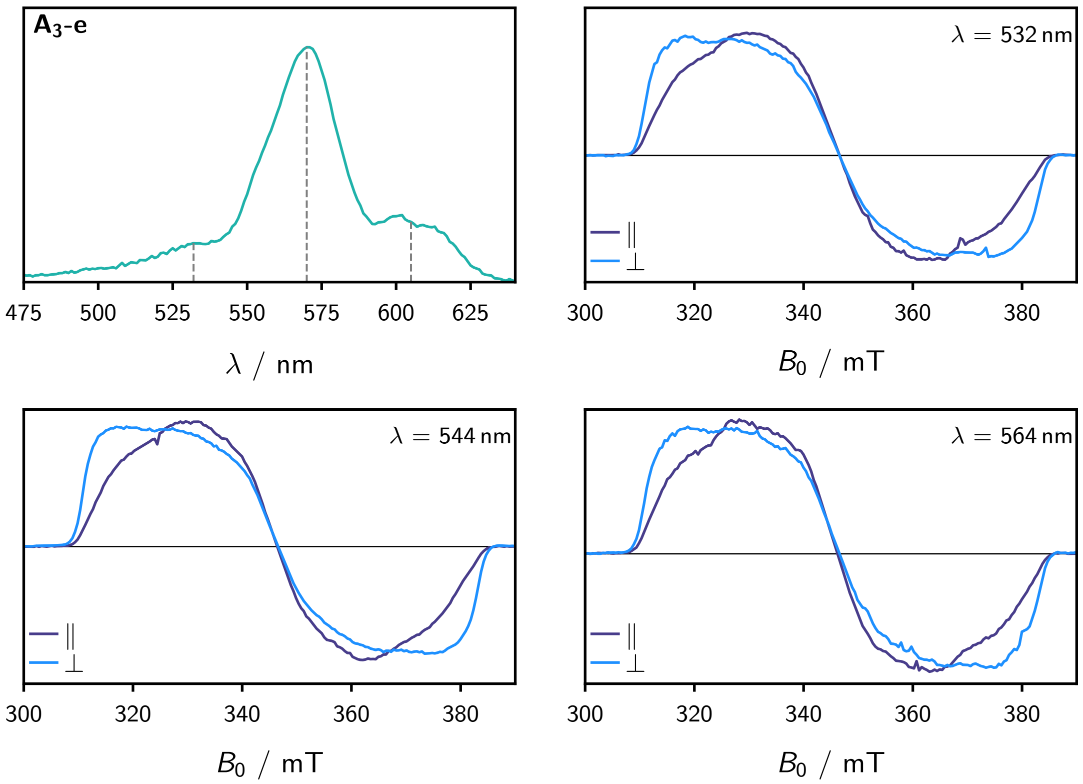

Figure S12: Electronic absorption spectrum, indicating the wavelengths chosen for MPS measurements on **A$_3$-e**, alongside the MPS spectra recorded with the light polarised parallel and perpendicular to the applied field.

S5 ENDOR

Given the ambiguous interpretation of the ENDOR spectra recorded at the *Z*/*X* canonical orientation, additional data were recorded at the *Y* orientation. As previous investigations have clearly demonstrated, if a change in principal axes orientations for the ZFS tensor occurs within a series, concomitant effects can be observed in the ENDOR spectra [8].
However, given the lack of distinguishing features in the ENDOR spectra, recorded at the *Y* orientation **Figure S13**, a conclusive and quantitative interpretation is almost impossible.

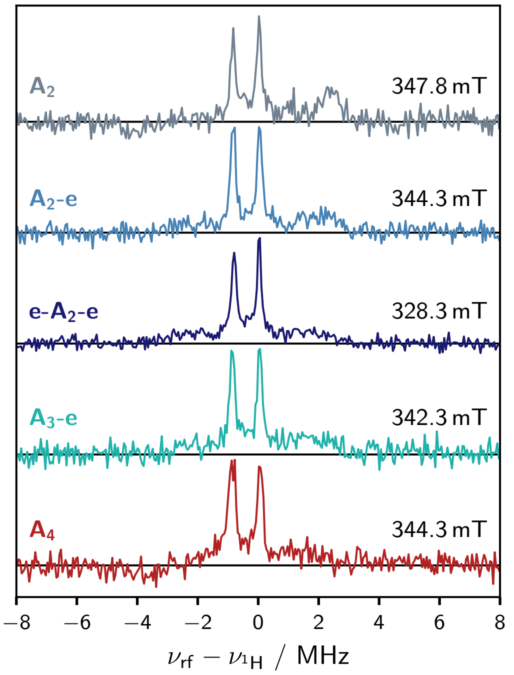

Figure S13: Proton Mims ENDOR spectra for all compounds recorded at the specified magnetic field position. The resonance fields correspond to primarily the *Y* canonical orientation of the $\boldsymbol{D}$-tensor. The narrow and intense lines located at ≈−1 MHz and 0 MHz, present in all spectra, correspond to the ^{19}F and ^{1}H Larmor frequencies, respectively.

S5.1 A_2-e Nuclide Assignment

Given the presence of $^{1}\mathrm{H}$ and $^{19}\mathrm{F}$ nuclei in the porphyrin systems and the similar nuclear g values, it is expected that the ENDOR spectra contain peaks from both nuclides. For the **A_2-e** system, a Gaussian fitting of multi-frequency ENDOR recorded at both the Z^+ and Z^- canonical orientations was performed. The results are presented in **Figure S14**.

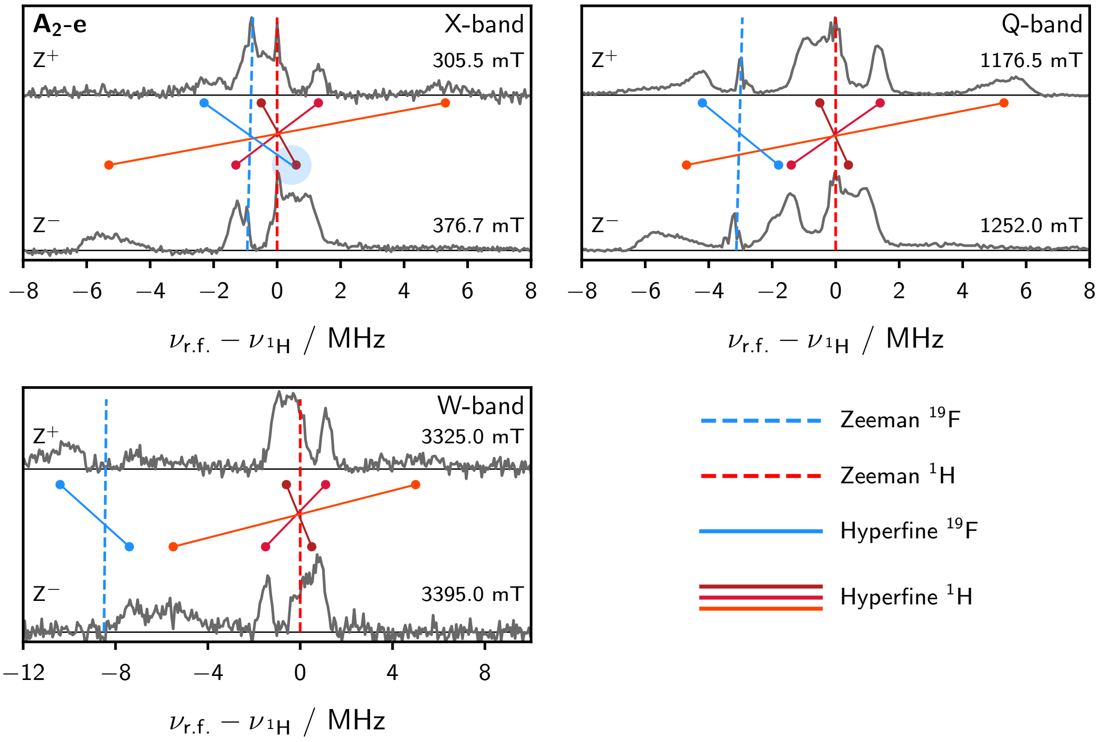

Figure S14: Mims ENDOR spectra acquired at Z^+ and Z^- at the X-band (*top-left*), Q-band (*top-right*), and W-band (*bottom-left*) for $\mathbf{A_2}$**-e**, alongside centre peak positions obtained by Gaussian fitting. The positions of the Larmor peaks are indicated by the blue and red broken lines for ^{19}F and ^{1}H, respectively. The assignment of peaks is indicated by the colour of the deconvolution marker. At the X-band, the ^{19}F high-field peak is obscured by the intense proton peak and its position is approximately indicated by the faded marker.

S6 DFT Calculations—Structures and Spin Densities

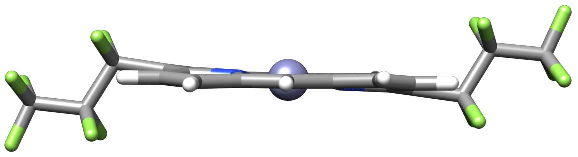

(a) e-A_2-e

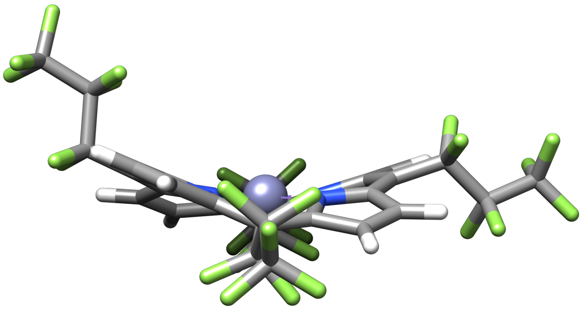

(b) A_4

Figure S15: Comparison of DFT optimised structures obtained for **e-**$\mathbf{A_2}$**-e** (least distorted) and $\mathbf{A_4}$ (most distorted).

To quantify the extent of out-of-plane distortions present in the systems, the mean absolute perpendicular displacements from an optimised mean plane are presented in **Table S6**. To aid visualisation, the structures for the most planar (**e-**$\mathbf{A_2}$**-e**) and most distorted ($\mathbf{A_4}$) structures are presented in **Figure S15**. The best-fit plane was obtained for minimizing the mean squared perpendicular displacement from the plane to the core porphyrin ring atoms (C, N and Zn).

Table S6: Mean of the absolute values for the perpendicular distance of point to plane ($\bar{r}_{\perp}$) compared with the ZFS D-value. Descending the table, we observe both an increase in distortion and a decrease in the D-value. However, the $|E/D|$ values appear to be uncorrelated with the extent of distortion.

	$\bar{r}_{\perp}$/Å	$\lvert D_{\mathrm{exp}}\rvert$/MHz	$\lvert E/D\rvert$
**e-A$_2$-e**	0.0479	1074	0.08
**A$_2$-e**	0.0864	1033	0.27
**A$_2$**	0.1116	1017	0.32
**A$_3$-e**	0.1489	1000	0.20
**A$_4$**	0.3336	967	0.28

References

[1] MATLAB; *version 9.5 (R2018b)*; The MathWorks Inc: Natick, Massachusetts, United States, **2018**.

[2] Stoll, S.; Schweiger, A. EasySpin, A Comprehensive Software Package for Spectral Simulation and Data Analysis in EPR. *J. Magn. Reson.*, **2006**, 178, 42–55.

[3] Stoll, S.; *Spectral Simulations in Solid-State Electron Paramagnetic Resonance*, PhD Thesis no. 15059, ETH Zürich, **2003**.

[4] Neese, F. The ORCA Program System. *WIREs: Comput. Mol. Sci.*, **2012**, 2, 73.

[5] Neese, F. Software Update: The ORCA Program System, Version 4.0. *WIREs: Computs. Mol. Sci.*, **2017**, 8, 1327.

[6] TURBOMOLE V6.1, **2009**, a development of University of Karlsruhe and Forschungszentrum Karlsruhe GmbH, 1989–2007, TURBOMOLE GmbH, since 2007; available from http://www.turbomole.com.

[7] Timmel, C. R.; *Magnetic Field Effects on Radical Pair Reactions*, DPhil Thesis, University of Oxford, **1998**.

[8] Tait, C. E.; Neuhaus, P.; Anderson, H. L.; Timmel, C. R. Triplet State Delocalization in a Conjugated Porphyrin Dimer Probed by Transient Electron Paramagnetic Resonance Techniques. *J. Am. Chem. Soc.*, **2015**, 137, 6670–6679.

[9] Tait, C. E.; Neuhaus, P; Peeks, M. D.; Anderson, H. L.; Timmel, C. R. Transient EPR Reveals Triplet State Delocalization in a Series on Cyclic and Linear π-Conjugated Porphyrin Oligomers. *J. Am. Chem. Soc.*, **2015**, 137, 8284–8293.

[10] Richert, S.; Bullard, G.; Rawson, J.; Angiolillo, P. J.; Therien, M. J.; and Timmel, C. R. On the Importance of Electronic Symmetry for Triplet State Delocalisation. *J. Am. Chem. Soc.*, **2017**, 139, 5301–5304.
